# An orally bioavailable pan-αv/α5β1 integrin antagonist prevents aggressive prostate cancer progression via suppressing both oncogenic signals and CD47-mediated immune escape

**DOI:** 10.1186/s12943-026-02686-7

**Published:** 2026-05-13

**Authors:** Yanlun Gu, Bingqi Dong, Xia Teng, Lin Chen, Yang Yang, Xiaojiao Sun, Song Song, Binzhi Qian, Yufang Sun, Jixin Zhang, Zhigang Zheng, Yuke Chen, Yimin Cui, Zhuona Rong, Xiaocong Pang

**Affiliations:** 1 Department of Pharmacy, Peking University First Hospital, Xishiku Street, Xicheng District, Beijing, 100034 China; 2 State Key Laboratory of Natural and Biomimetic Drugs, School of Pharmaceutical Sciences, Peking University, XueYuan Road 38, HaiDian District, Beijing, 100191 China; 3 Institute of Clinical Pharmacology, Peking University First Hospital, Xueyuan Road 38, Haidian District, Beijing, 100191 China; 4 Beijing Key Laboratory of Clinical Pharmacology Translation of Innovative Drugs, Peking University First Hospital, Xishiku Street, Xicheng District, Beijing, 100034 China; 5 Department of Gastrointestinal Surgery, Peking University First Hospital, Xishiku Street, Xicheng District, Beijing, 100034 China; 6 Edinburgh Cancer Research UK Centre, Institute of Genetics and Cancer, University of Edinburgh, Edinburgh, UK; 7 Fudan University Shanghai Cancer Center, Department of Oncology, Shanghai Medical College, Center for Integrative Spatial-Omics Research, The Human Phenome Institute, Zhangjiang-Fudan International Innovation Center, Fudan University, Shanghai, China; 8 Department of Pathology, Peking University First Hospital, Xishiku Street, Xicheng District, Beijing, 100034 China; 9 Department of Urology, Peking University First Hospital, Xishiku Street, Xicheng District, Beijing, 100034 China; 10 Department of Urology, Affiliated Cancer Hospital of Zhengzhou University, Henan Cancer Hospital, Zhengzhou, 450008 China

## Abstract

**Background:**

Advanced prostate cancer (PCa), particularly enzalutamide-resistant, bone-metastatic castration-resistant prostate cancer (CRPC) and neuroendocrine prostate cancer (NEPC), remains an intractable clinical challenge due to oncogenic crosstalk across multiple pathways and tumor microenvironment (TME) heterogeneity. Given these unmet clinical needs, novel targeted therapeutics are urgently required to improve advanced PCa patient outcomes.

**Methods:**

In this study, we examined the expression of integrin subtypes, particularly pan-αv integrins and α5β1, in prostate cancertumor samples and cell lines using immunohistochemistry (IHC) and Western blotting. Based on rational drug design, we synthesized a first-in-class, orally bioavailable, non-RGD pan-αv/α5β1 integrin antagonist, C19-9N, and characterized it through homology modeling, molecular docking, surface plasmon resonance (SPR), and microscale thermophoresis (MST). The antitumor activity of C19-9N was evaluated in vitro (via assays of cell viability, migration and invasion) and in vivo using multiple models, including subcutaneous xenografts of CRPC, bone-metastatic xenograft models established by intratibial and intracardiac injection, and patient-derived xenografts (PDXs) of NEPC. The in vivo safety and pharmacokinetic profile of C19-9N were also assessed. Mechanistically, single-cell RNA sequencing (scRNA-seq) was employed to uncover the regulatory effects of C19-9N on infiltrating immune cells within the tumor microenvironment, which were further validated through multiplex immunofluorescence and bone marrow-derived macrophage-mediated phagocytosis assays.

**Results:**

C19-9N targeting pan-αv and α5β1 integrin circumvented potential compensatory resistance mediated by integrin subtype switching. C19-9N disrupted extracellular matrix (ECM)-integrin biochemical and mechanical signaling, thereby suppressing cancer stem cell (CSC) self-renewal and epithelial-to-mesenchymal transition (EMT). Mechanistically, C19-9N inhibited PI3K/AKT and STAT3 signaling pathways to block alternative splicing of AR-V7, and modulated Survivin and c-Myc to enhance enzalutamide sensitivity. Additionally, it remodeled TME by repolarizing tumor-associated macrophages (TAMs) toward a pro-inflammatory phenotype and downregulating CD47-mediated immune escape. In preclinical models, C19-9N overcame enzalutamide resistance in CRPC xenografts, suppressed bone metastatic progression, and exhibited superior efficacy to platinum/taxane therapies in NEPC.

**Conclusion:**

Collectively, by co-targeting oncogenic drivers and TME vulnerabilities, C19-9N heralds a transformative therapeutic paradigm with profound clinical potential for aggressive PCa.

**Supplementary Information:**

The online version contains supplementary material available at 10.1186/s12943-026-02686-7.

## Introduction

Prostate cancer (PCa) is the most prevalent male malignancy globally, with rising incidence risk driving a growing morbidity burden and sustained mortality rate worldwide. While early-stage PCa is now manageable with improved clinical strategies, advanced disease remains uniformly lethal [1, 2], primarily due to inherent tumor heterogeneity, aggressive subtype biology, and frequent treatment resistance in metastatic settings [3, 4]. Aberrant androgen receptor (AR) signaling is the central molecular driver of PCa initiation and progression, and this dependency persists even in advanced disease [5]. This reliance on AR signaling persists even in advanced disease, as exemplified by castration-resistant prostate cancer (CRPC)-a subtype defined by clinical relapse in metastatic PCa patients following primary androgen ablation therapy [6]. A key contributor to this poor prognosis is bone metastasis, an endemic complication of advanced PCa that not only accounts for most disease-related mortality but also severely impairs patient quality of life, exacerbating the overall morbidity burden of the disease. About 10—17% of patients with CRPC evolve as neuroendocrine prostate cancer (NEPC), characterized by low or absent AR expression, independence of AR signaling and gain of a neuroendocrine phenotype, which has the poorest outcome, with a median survival of less than one year [7, 8]. Given these unmet clinical needs, novel targeted therapeutics are urgently required to improve advanced PCa patient outcomes.

Alterations in the integrin family of adhesion receptors throughout prostate tumorigenesis are closely associated with the progression of aggressive disease behaviors and the acquisition of therapeutic resistance [9, 10]. Integrins are a family of transmembrane glycoprotein receptors composed of α and β subunits that form heterodimers. Their core function is to mediate adhesion between cells and the extracellular matrix (ECM), as well as cell-to-cell interaction. Integrins are divided into four classes according to cellular expression or ligand identity [11]. The abnormal expression or activation of Arg-Gly-Asp (RGD)-binding integrins [12, 13] contribute to PCa progression and metastasis by activating downstream signaling molecules of cell proliferation, adhesion and invasiveness, including MAPK, FAK, SRC, AKT and JNK [14]. Cell adhesion-mediated drug resistance (CAMDR), driven in part by ECM-integrin axis, is a fundamental mechanism underlying therapeutic failure in PCa [15]. Integrin αvβ3 is usually present at very low levels in normal human prostate cells but is highly expressed in PCa cells and metastasis, and its abnormal expression is associated with PCa aggressive phenotypes development via promoting invasion and adhesion of cancer cells [16, 17]. The expression of αvβ3 is up-regulated in primary epithelial cells from PCa [18], and αvβ3 is found in exosomes shed by prostate epithelial cells in the plasma of PCa patients, leading to metastatic lesions, mainly bone [19]. αvβ3 receptor in PCa cells is required not only for tumor growth, but also for migration to bone matrix and determines bone lesion development [20]. In NEPC, exosomes expressing the αvβ3 integrin contribute to neuroendocrine differentiation through a GPI-anchored receptor, NgR2 [21]. Integrin αvβ6 via activation of JNK1 increases nuclear localization and activity of AR and consequently promotes up-regulation of Survivin [13]. In addition, αvβ6 from cancer cells could transfer to monocytes through exosomes to regulate M2 polarization in PCa progression [22]. Trop-2 modulates β1 integrin-dependent adhesion and metastatic dissemination of PCa cells through relocalization of α5β1 integrin [23]. Knockdown of integrin β1 resulted in suppression of full-length AR (AR-FL) and AR splice variant-7 (AR-V7) expression in 22RV1 prostate cancer cells, which suggested integrin β1 regulates AR-V7 splicing, in addition, SRC activation could promote AR-V7 splicing and protein stability [24]. In our previous study, FN1-α5β1-SRC signaling cascade drove anti-androgen resistance in bone metastatic PCa, which linked to macrophage induced wound-healing response [25]. Therefore, targeting RGD-binding integrins is expected to achieve a dual effect of combating drug resistance to androgen-targeted therapies and bone metastases.

However, most current RGD-based small-molecule antagonists targeting the αv integrin family function as partial agonists [11]. These agents induce profound conformational changes in integrins, triggering paradoxical cell adhesion and angiogenesis—critical barriers to effective anti-tumor therapy [11]. To address this unmet need, non-peptide RGD-mimetic integrin ligands with high affinity and potent anti-tumor efficacy have emerged as a pivotal focus for the development of integrin targeted therapeutics [18]. In our prior work, we identified C19—9, a novel non-RGD pure antagonist that blocks αvβ3 and exhibits therapeutic potential in CRPC [26]. However, most prior investigations, including our own preliminary work, have focused on single integrin subtype, failing to account for the inherent functional redundancy and compensatory crosstalk that characterize integrin signaling networks. Herein, we report the structural optimization of C19—9, leading to the development of C19-9N as an orally bioavailable pan-αv integrin and α5β1 antagonist. C19-9N not only potently enhances the anti-tumor activity of the second-generation anti-androgen enzalutamide but also abrogates bone metastasis and suppresses neuroendocrine transformation, two aggressive phenotypes driving treatment failure in advanced PCa. Our findings establish C19-9N as a promising monotherapy or combination partner for the treatment of aggressive PCa, addressing key limitations of current standard-of-care strategies.

## Results

### Identification of C19-9N as pure pan-αv and α5β1 integrin antagonist blocking ECM-integrin interaction

To delineate integrin expression patterns during PCa progression, we performed immunohistochemical (IHC) analysis on a cohort of primary PCa (*n* = 10) and metastatic castration-resistant PCa (mCRPC, bone metastasis; *n* = 10) tissues. Integrin αv was the most prominently expressed integrin subunit across all samples, showing notably stronger expression in mCRPC tissues relative to prostate-specific membrane antigen (PSMA) (Figure S1A). In contrast, α2 and α5 showed moderate expression, while α6 was lowest among the α subunits analyzed. Among β subunits, β1, β3, and β5 were robustly higher expressed in both tissue types compared to normal prostate epithelium (*n* = 6), with β3 showing significantly higher expression in mCRPC compared to primary PCa.We further validated αv and α5 integrin protein expression in PCa cell lines spanning different progression stages and silencing of ITGAV results in compensatory upregulation of integrin α5. (Figure S1B-C), revealing partially complementary expression patterns of αv and α5 integrins. In addition, silencing αv or α5 via siRNA in CRPC 22RV1 cells significantly enhanced enzalutamide-induced apoptosis (Figure S1D), underscoring the contribution of these integrins to therapy resistance.

**Fig. 1:**
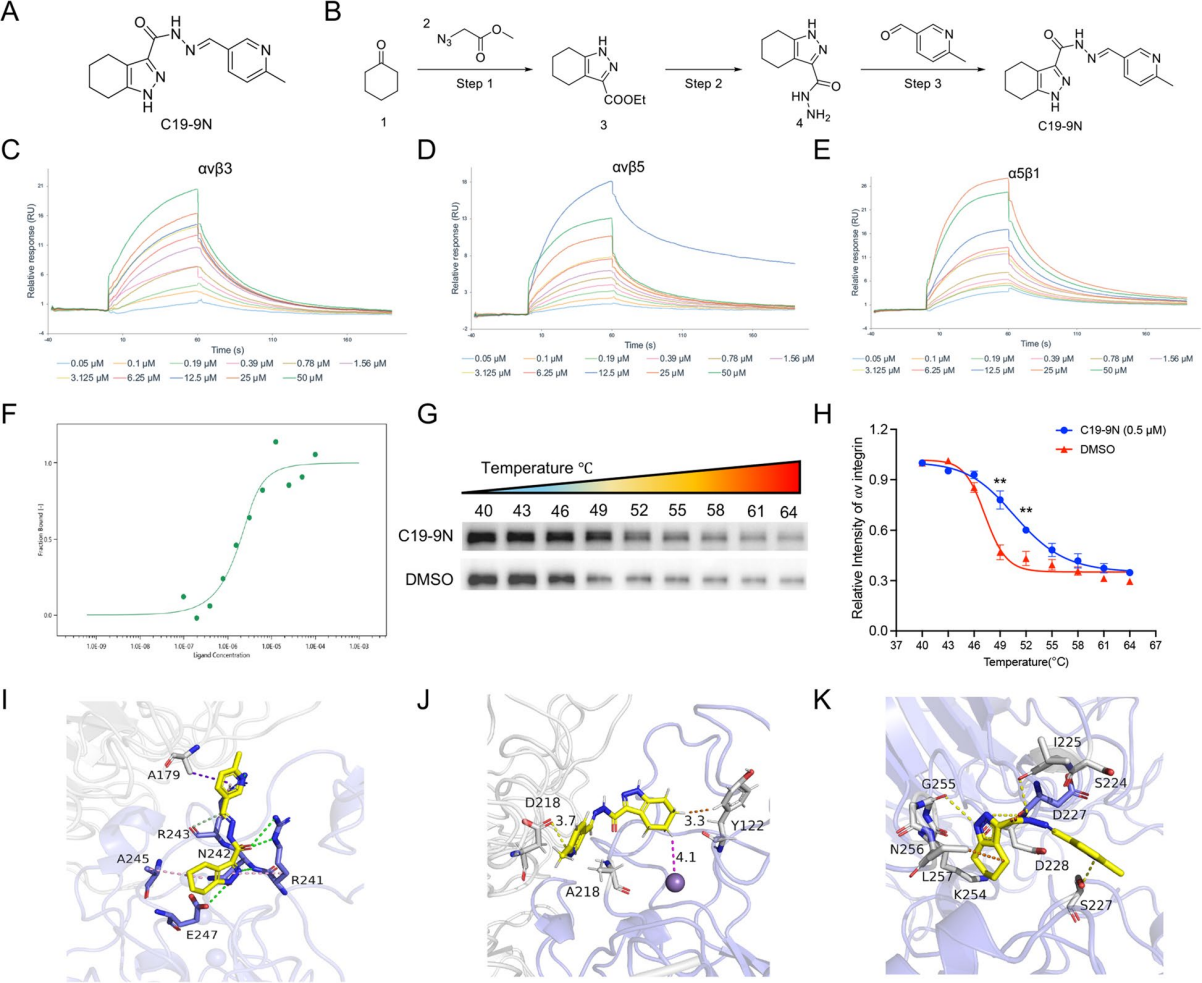
Identification of C19-9N as high affinity inhibitor of pan-αv/integrin α5β1. **A** The structure of C19-9N. **B** Synthesis route of C19-9N. **C**-**E** The affinity of C19-9N with αvβ3, αvβ5 and α5β1 measured by SPR method, respectively. **F** The affinity of C19-9N without Mn^2+^ measured by MST method. **G**-**H** The thermal stability results of integrin αv by CETSA-WB. **I**-**K** Molecular docking using Glide (Schrödinger) against αvβ5, αvβ3, and α5β1, respectively

In our prior work, we identified C19—9 as a selective αvβ3 antagonist that suppresses PCa tumorigenesis by inhibiting cancer cell proliferation, adhesion, and migration [26]. To achieve concurrent targeting of pan-αv integrins and α5β1 (key mediators of therapy resistance and metastasis), we rationally modified the structure of C19—9 by substituting its aromatic ring with a nitrogen-containing heterocycle (pyridine scaffold; Fig. 1A), a design intended to optimize pocket occupancy across multiple integrin subtypes. The synthesis route of the resulting analog, C19-9N, is detailed in Fig. 1B and Supplementary Figure S2A-B. Notably, the introduction of the pyridine heterocycle and a methoxy group enhanced fit within the binding pockets of pan-αv integrins and α5β1, as supported by binding affinity assays: C19-9N exhibited high affinity for αvβ3 (Kd = 0.55 μM), αvβ5 (Kd = 0.61 μM), α5β1 (Kd = 0.88 μM), αvβ6 (Kd = 0.41 μM) and αvβ8 (Kd = 0.86 μM) (Fig. 1C-E and Figure S2C-D). Microscale thermophoresis (MST) was also applied to further validate the binding affinity of C19-9N. As a result, the KD value of C19-9N was 0.58 μM in the absence of Mn^2+^, different from RGD ligands, which were mostly divalent cation-dependent and C19-9N did not depend on Mn^2+^ induced conformational changes (Fig. 1F). Fibronectin, a key extracellular matrix component, triggers outside-in integrin signaling through its divalent cation-dependent binding αvβ3. Specifically, the Mn^2+^-facilitated interaction between fibronectin and αvβ3 was blocked by C19-9N (Figure S2E-F). We further performed CETSA on 22RV1 endogenously expressing the target integrin αv. Treatment with C19-9N resulted in a significant thermal stabilization of integrin αv, directly demonstrating intracellular target engagement(Fig. 1G-H).

To elucidate the structural basis of C19-9N’s pan-integrin binding, we performed molecular docking using Glide (Schrödinger) against available X-ray crystal structures of αvβx subtypes and α5β1. Since no αvβ5 crystal structure has been reported, we generated a homology model of human αvβ5 (Figure S3). Ramachandran plot analysis confirmed the model’s reliability, with 85.59% of residues residing in allowed regions (Figure S3A-B). The αvβ5 model shared 84.35% sequence identity with the template structure, and its overall fold was consistent with the template, validating its use for docking (Figure S3C-D). The predicted binding mode of C19-9N to αvβ5 (Fig. 1I) revealed high affinity engagement, achieved via the formation of six conventional hydrogen bonds with β5 residues (R241, N242, R243, and E247), and a pi-sigma hydrophobic interaction with αv residue A179.

For αvβ3 (PDB: 1L5G), C19-9N’s pyridine nitrogen formed a hydrogen bond with D218, the cyclohexyl moiety engaged in hydrophobic contact with Y122, and a metal coordination bond was observed with the Mn^2^⁺ ion in the integrin’s metal ion-dependent adhesion site (MIDAS; Fig. 1J). In the αvβ6-C19-9N complex, four hydrogen bonds were formed with T314, Q317, R248, and D219, alongside hydrophobic interactions with I216 (Figure S3E). For α5β1, C19-9N established four hydrogen bonds with G255, D277, I225, and S227, plus hydrophobic contacts with L257 (Fig. 1K). Collectively, these diverse interactions, encompassing hydrogen bonds, hydrophobic contacts, and metal coordination, provide a structural rationale for C19-9N’s high affinity for pan-αv integrins and α5β1, supporting its design as a multi-targeted integrin antagonist.

### C19-9N as a potent inhibitor of prostate cancer cell growth, migration, and invasion in vitro

**Fig. 2:**
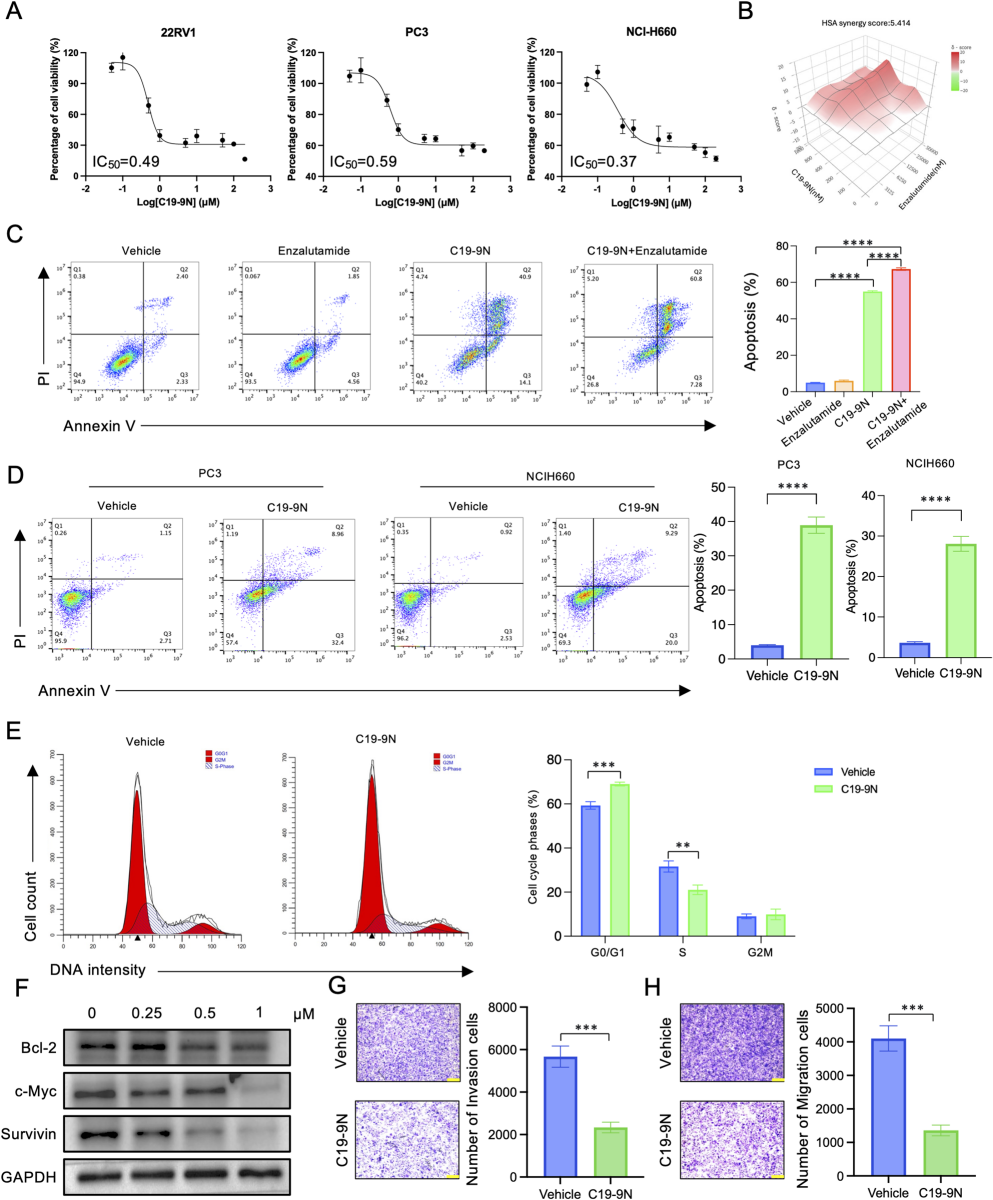
C19-9N suppresses PCa tumorigenesis by inhibiting cancer cell growth, migration, and invasion in vitro. **A** The CCK8 assay of evaluation of anti-tumor proliferation ability of C19-9N on 22RV1, PC-3 and NCI-H660 at 48h. **B** The synergistic effect on inhibition of 22RV1 cell proliferation of C19 and enzalutamide. **C** C19-9N enhances enzalutamide-induced apoptosis in 22RV1. Representative flow cytometry dot plots showing apoptotic cells (Annexin V-FITC⁺/PI⁻ or Annexin V-FITC⁺/PI⁺) in cells treated with vehicle, enzalutamide, C19-9N, or C19-9N combined with enzalutamide. Right panel: Quantitative analysis of apoptosis rate. **D **C19-9N induces apoptosis in PC3 and NCI-H660. Representative flow cytometry dot plots showing apoptotic cells (Annexin V-FITC⁺/PI⁻ or Annexin V-FITC⁺/PI⁺) in cells treated with enzalutamide and C19-9N. Right panel: Quantitative analysis of apoptosis rate. **E** The effect of C19-19N on cell cycle analyzed by DNA content staining in 22RV1. Left panels: Representative flow cytometry histograms (DNA intensity) showing cell cycle distribution (G0/G1, S, G2/M phases) in vehicle or C19-9N treated cells. Right panel: Quantitative analysis of cell cycle phase percentages. **F** Western blot analysis of pro-survival proteins. Representative blots showing the expression of Bcl-2, c-Myc, and Survivin in cells treated with increasing concentrations of C19-9N (0–1 μM). **G-H** The impact of C19-9N on prostate cancer cell motility and invasiveness via transwell migration and invasion assays. Scale bar = 200 μm. Data are expressed as mean ± SD, *P-value < 0.05, **P-value <0.01, ***P-value < 0.001

To investigate the anti-tumor activity of C19-9N in prostate cancer, we conducted in vitro evaluations using diverse cell line models representing distinct molecular subtypes. As shown in Fig. 2 A, C19-9N significantly inhibited the proliferation of AR-positive CRPC (22RV1), AR-negative mCRPC (PC3), and NEPC (NCI-H660) cells, with IC_50_ values of 0.49 μM, 0.59 μM, and 0.37 μM, respectively. This broad anti- proliferative efficacy highlights C19-9N’s potential to target PCa subsets regardless of AR expression or neuroendocrine differentiation. Given the clinical challenge of enzalutamide resistance in CRPC, we next explored combinatorial effects between C19-9N and enzalutamide via three-dimensional synergy analysis in the 22RV1 cell line. As illustrated in Fig. 2B, the combination exhibited significant cooperativity, with an HSA synergy score of 5.414. Mechanistically, C19-9N monotherapy induced robust apoptosis, whereas enzalutamide alone failed to elicit a meaningful apoptotic response (Fig. 2C). Notably, co-treatment with C19-9N and enzalutamide further enhanced apoptotic activity compared to C19-9N monotherapy in 22RV1 cells, confirming a synergistic role in promoting cancer cell death and supporting the translational potential of this combination strategy. As shown in Figure S4A, we further validated this synergistic pro-apoptotic effect in the C4—2B cell model, a clinically relevant, enzalutamide-resistant line with AR dependency. Moreover, consistent with our broader mechanistic investigation, C19-9N alone was also found to significantly induce apoptosis in AR-null models, including PC3 and NCI-H660 (Fig. 2D). These results collectively indicate that C19-9N exerts pro-apoptotic activity across diverse prostate cancer subtypes, regardless of AR status, thereby highlighting its potential as a broadly effective therapeutic agent.

Then, Cell cycle analysis in 22RV1, PC3, and NCI-H660 cell lines via flow cytometry revealed a consistent arrest in the G0/G1 phase following C19-9N treatment. This was evidenced by a marked increase in the proportion of G0/G1 phase cells, accompanied by a significant reduction in S phase cells, with no appreciable change observed in the G2/M phase population (Fig. 2E and Supplementary Figure S4B). Consistent with these phenotypes, western blot analysis revealed that C19-9N modulated the expression of key oncogenic markers involved in apoptosis and cell cycle regulation, including Mcl-1, Bcl-2, c-Myc, and Survivin (Fig. 2F, Figure S4C), indicating that it targeted critical oncogenic pathways. Notably, C19-9N suppressed Survivin expression in a concentration-dependent manner. Survivin, which is regulated by αvβ3, has been implicated in conferring resistance to hormone therapy [27, 28]. In addition, we assessed the impact of C19-9N on prostate cancer cell motility and invasiveness via transwell migration and invasion assays. As shown in Fig. 2G-H, C19-9N treatment significantly inhibited both migration and invasion of prostate cancer cells. Collectively, these findings demonstrate that C19-9N exerts multi-faceted anti-tumor effects through inducing apoptosis, arresting cell cycle progression, and suppressing integrin-mediated adhesion and migration, thus providing a strong rationale for its development against aggressive, therapy-resistant PCa.

### C19-9N inhibits prostate cancer cell stemness and EMT process

**Fig. 3:**
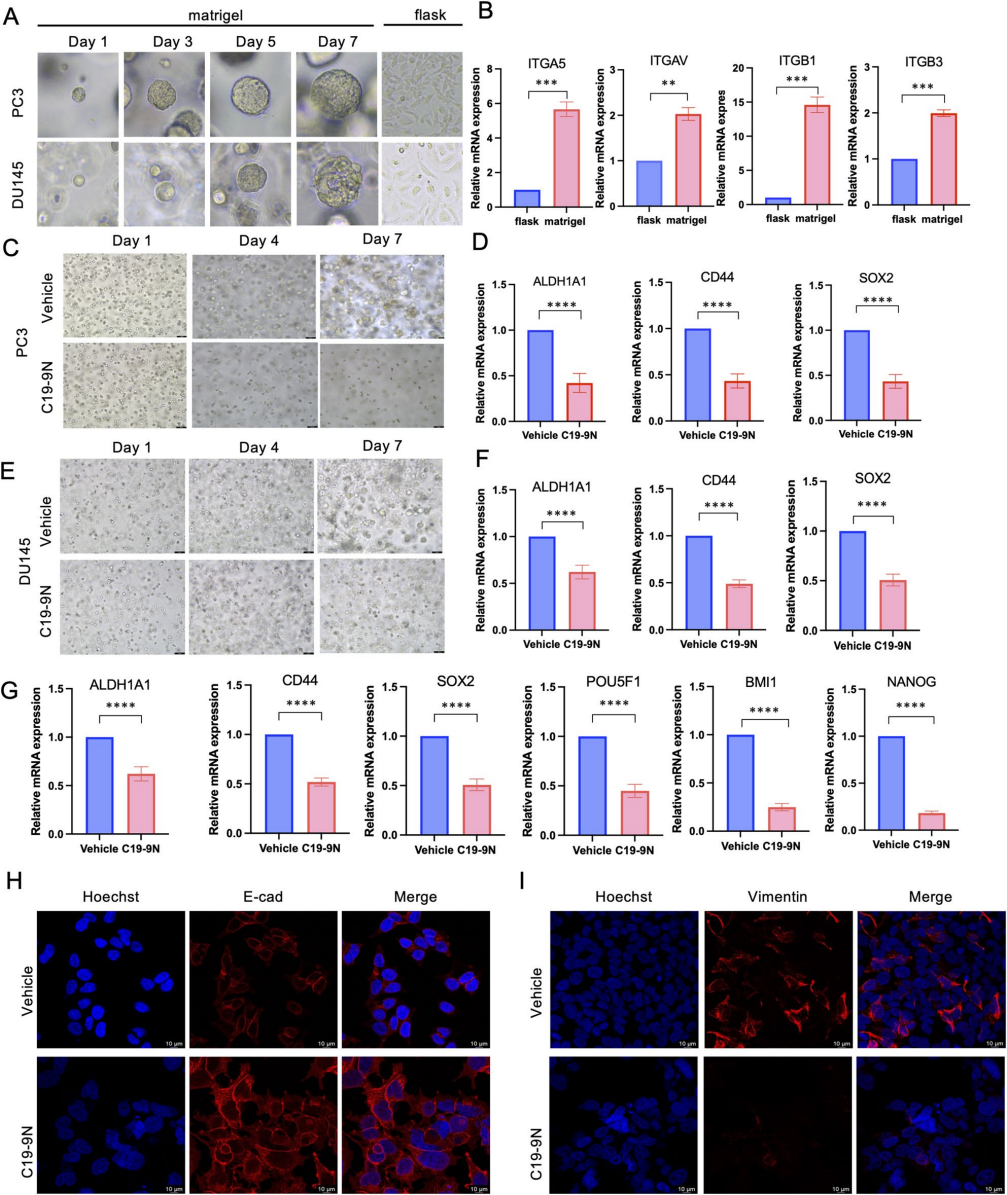
C19-9N inhibits CSC properties and reverses EMT in PC3 cells. **A** Matrigel-induced CSC-like morphology. Representative bright-field images of PC3 cells cultured in Matrigel (top row, Days 1-7) or standard flask (bottom right, Day 7). Matrigel culture promoted a rounded, CSC-like spherical phenotype. **B** Integrin mRNA upregulation in Matrigel. Quantitative analysis of ITGA5 (α5), ITGB1 (β1), ITGB3 (β3), ITGAV (αv) mRNA expression in PC3 cells cultured in flask vs. Matrigel. **C,E** C19-9N inhibits sphere formation. Representative images of PC3 and DU145 spheres (Day 1, 4, 7) treated with Vehicle or C19-9N. Scale bar = 100 μm.C19-9N reduced sphere size/number (CSC self-renewal capacity). **D,F** C19-9N downregulates stemness genes. Quantitative analysis of stemness-associated (SOX2, CD44, ALDH1A1) mRNA expression. **G** C19-9N reduces the expression of key stemness markers in DU145-DR cells. Quantitative analysis of stemness-associated (ALDH1A1, CD44, SOX2, POU5F1, BMI1, NANOG) mRNA expression. **H** C19-9N increases epithelial marker E-cadherin. Immunofluorescence staining (Hoechst: nuclear, E-cad: E-cadherin) of PC3 cells. Scale bar = 10 μm. **I** C19-9N decreases mesenchymal marker Vimentin. Immunofluorescence staining (Hoechst: nuclear, Vimentin: mesenchymal marker) of PC3 cells. Scale bar = 10 μm. Data are expressed as mean ± SD, *P-value < 0.05, **P-value <0.01, ***P-value < 0.001

Integrins are key transmembrane receptors that transduce chemical and mechanical signals, acting as central mediators of ECM-cell crosstalk to regulate intracellular signaling cascades [29]. Given that ECM stiffness is a critical biomechanical cue CSC self-renewal and tumor progression in PCa, we sought to mimic ECM-induced biomechanical signaling using Matrigel (a native ECM surrogate) as previously described [30]. PC3 and DU145 prostate cancer cells cultured in Matrigel exhibited a rounded, CSC-like morphology (Fig. 3A) and significantly upregulated mRNA expression of αvβ3 and α5β1 integrins (Fig. 3B), which supporting that ECM biomechanics promotes integrin activation and stemness-associated phenotypic reprogramming [29]. To assess whether C19-9N targets integrin-dependent stemness, we performed sphere formation assays (a gold standard for CSC self-renewal capacity). C19-9N-mediated blockade of αvβ3 and α5β1 not only reduced sphere size and number but also downregulated the expression of stemness-associated genes (SOX2, CD44 and ALDH1A1) in PC3 and DU145 cell lines (Fig. 3C-F), demonstrating that C19-9N directly suppresses prostate CSC properties via integrin inhibition. The sensitivity of DU145 cells to docetaxel was significantly reduced under a stiff matrix environment. When cultured in Matrigel (simulating a hard matrix), the IC_50_ value increased to 32.19 nM compared to 6.11 nM in the standard flask culture (Figure S5A). To further investigate this effect in a clinically relevant context of therapy resistance, we established a docetaxel-resistant DU145 subline (DU145-DR) through chronic exposure to increasing concentrations of docetaxel. This model exhibits hallmark features of drug resistance, including enhanced expression of stem cell-associated markers [31, 32]. Critically, in DU145-DR cells, C19-9N treatment significantly reduced the expression of key stemness markers (e.g., ALDH1A1, CD44, SOX2, POU5F1, BMI1, NANOG) (Fig. 3G), confirming that its anti-CSC activity is maintained even in a chemoresistant background.

EMT is tightly coupled to integrin signaling and CSC phenotypes, serving as a key driver of tumor invasiveness and therapy resistance [33]. Consistent with its inhibitory effects on cell migration and invasion, C19-9N treatment reversed EMT in PC3 and DU145-DR cells: it decreased the mesenchymal marker Vimentin and increased the epithelial marker E-cadherin (Fig. 3H-I, Supplementary Figure S5B-C). Together, these findings establish a mechanistic link between integrin inhibition, CSC suppression, and EMT reversal, thereby defining the multi-pronged anti-tumor mechanism of C19-9N in aggressive PCa.

### C19-9N exerts a robust effect on AR-V7 alternative splicing

**Fig. 4:**
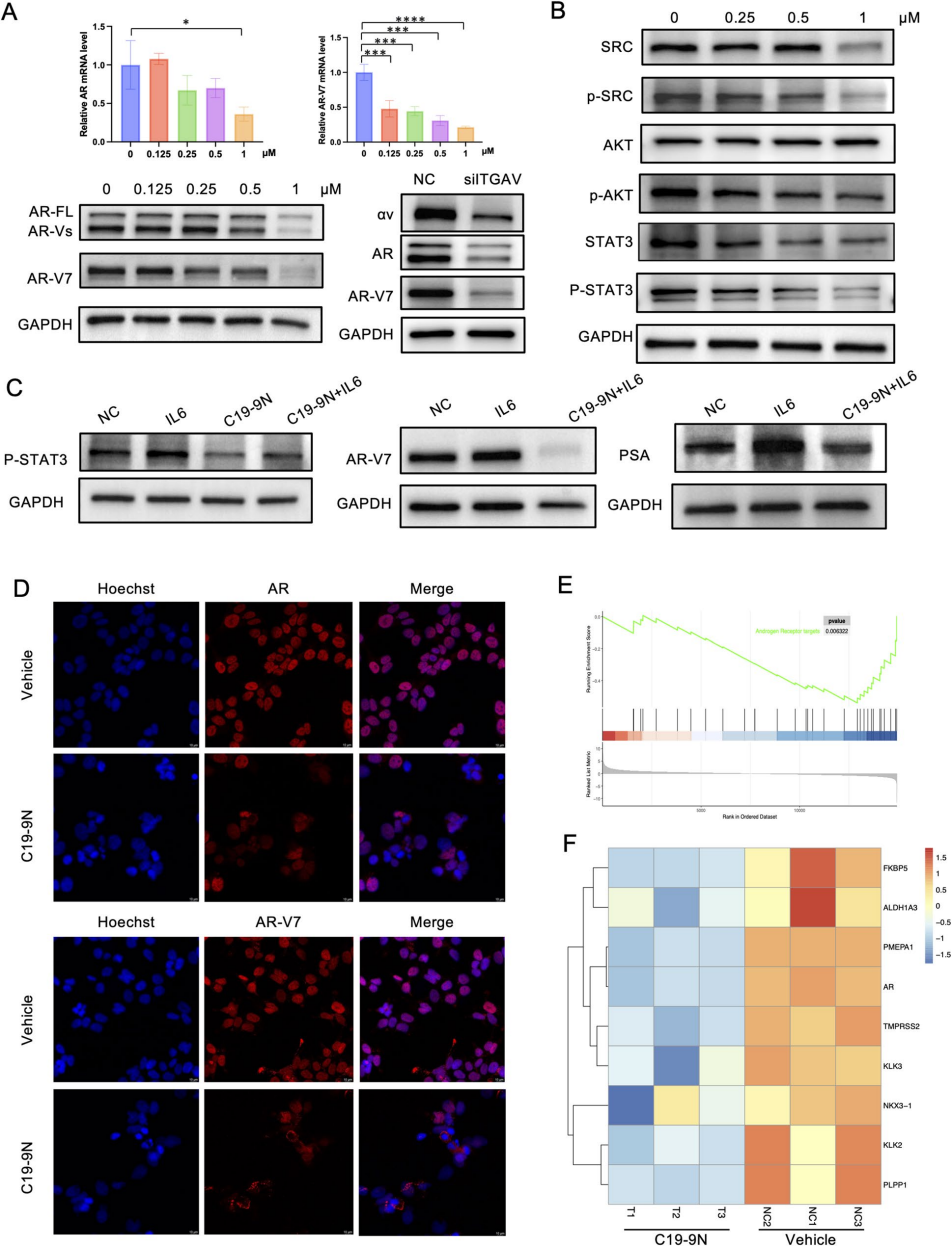
C19-9N exerts a robust effect on AR-V7 alternative splicing. **A** C19-9N and ITGAV knockdown downregulate AR-FL/AR-V7. Top: Quantitative analysis of AR-FL and AR-V7 mRNA expression in cells treated with C19-9N. Bottom: Representative western blots showing AR-FL, total AR-Vs, AR-V7 protein levels (C19-9N treatment, left; ITGAV knockdown, right). **B** C19-9N inhibits SRC, AKT, STAT3 phosphorylation. **C** C19-9N abrogates IL-6-induced STAT3 activation and suppresses downstream AR-V7 and PSA expression. Left: Western blot analysis of P-STAT3. Cells were overnight treated with vehicle (NC) or C19-9N, followed by stimulation with 10 ng/mL IL-6 for 30 minutes. Subsequently, cellular proteins were extracted to capture the rapid phosphorylation cascades in each group. Middle and Right: Western blot analysis of downstream effectors AR-V7 and PSA. cells were exposed to vehicle (NC), IL-6, or C19-9N+IL6 for 48 hours. GAPDH served as the loading control for all assays. **D** C19-9N blocks nuclear localization of AR-FL/AR-V7. C19-9N reduced overall AR/AR-V7 levels and nuclear translocation. Scale bar = 10 μm. **E** GSEA of AR-regulated gene signature (p = 0.0063). **F** C19-9N reduces mRNA expression of AR target genes. Heatmap of AR target genes expression. Data are expressed as mean ± SD, *P-value < 0.05, **P-value <0.01, ***P-value < 0.001

AR-V7 is a well-established major driver of enzalutamide resistance in castration-resistant prostate cancer (CRPC), representing a critical unmet clinical challenge [34]. The 22RV1 cell line is widely recognized and utilized as a classical and clinically relevant model of intrinsic enzalutamide resistance due to its constitutive expression of AR-V7 [35]. In 22RV1 cell line, we demonstrate that C19-9N exerts robust, dose-dependent downregulation of AR-V7 mRNA, whereas its effect on AR-FL mRNA is inconsistent with significant reduction only observed at the highest concentration (1 μM) (Fig. 4A). Consistently, C19-9N dose-dependently suppressed the protein expression of total AR variants (AR-Vs) and AR-V7, while AR-FL inhibition was pronounced solely at 1 μM (Fig. 4A). Furthermore, we validated this effect in the C4—2B model, where treatment with C19-9N (0.5 μM) significantly reduced the protein levels of both AR-FL and AR-V7 (Figure S5D), confirming the compound’s ability to target AR signaling in an additional clinically relevant context. Notably, western blot analysis confirmed that ITGAV knockdown recapitulated these effects, downregulating AR-FL, total AR-Vs, and AR-V7 expression (Fig. 4A), which supporting ITGAV targeting as a rational dual-targeting strategy for both AR-FL and AR-V7 in CRPC. Importantly, the partial suppression of AR-FL by C19-9N may underlie its mechanism of sensitizing cells to enzalutamide; by reducing AR-FL expression without complete abrogation, C19-9N may create a cellular state more susceptible to enzalutamide-mediated inhibition, rather than relying solely on full AR blockade.

Mechanistically, ITGAV knockdown or integrin αv blockade has been shown to downregulate SRC phosphorylation, a key event that modulates AR transcriptional activity and nuclear translocation [36, 37]. Additionally, transcription factor STAT3 promotes AR nuclear localization and transcriptional activity, while the PI3K/AKT/mTOR pathway activation is pivotal for enhancing AR-V7 alternative splicing [38]. Consistent with these prior findings, our results demonstrate that C19-9N significantly inhibits the expression of SRC and STAT3, as well as the phosphorylation of SRC, AKT, and STAT3 (Fig. 4B), key signaling intermediates that drive AR activation and AR-V7 generation. Furthermore, we investigated the impact of C19-9N on the IL-6/STAT3 axis, a critical pathway involved in resistance [39]. As shown in Fig. 4C, treatment with IL-6 induced a marked increase in P-STAT3 levels, which was effectively reversed by C19-9N in a dose-dependent manner. Additionally, C19-9N treatment significantly downregulated the protein expression of AR-V7 and its downstream target PSA, indicating that inhibition of this pathway contributes to the suppression of AR-V7 signaling. Given that ligand-activated AR dimerizes, translocates to the nucleus, and binds to androgen response elements (AREs) to regulate target gene expression, we performed immunofluorescence assays and observed that C19-9N treatment remarkably decreased both the nuclear localization and overall protein levels of AR-FL and AR-V7 (Fig. 4D and Figure S5E-F). To validate the broader impact of C19-9N on AR transcriptional activity, total RNA was extracted from 22RV1 cells treated with 0.5 μM C19-9N for 48 h and applied for RNA-Seq assay. Based on gene set enrichment analysis (GSEA), we found a signature of AR-regulated genes was significantly downregulated (*P* = 0.0063) (Fig. 4E). Complementing this genome-wide analysis, C19-9N treatment of 22RV1 cells (an AR-V7-positive CRPC model) significantly reduced the mRNA expression of well-characterized AR target genes, including TMPRSS2, KLK2, NKX3.1, and FKBP5 (Fig. 4F). Collectively, these data suggest C19-9N inhibits SRC/AKT/STAT3 signaling, leading to downregulation of AR-FL/AR-V7 expression, blockade of their nuclear translocation, and suppression of AR transcriptional activity. This multi-layered mechanism provides a compelling preclinical rationale for C19-9N as a therapeutic strategy to overcome enzalutamide resistance in AR-V7-positive CRPC.

### C19-9N overcomes enzalutamide resistance in vivo

**Fig. 5:**
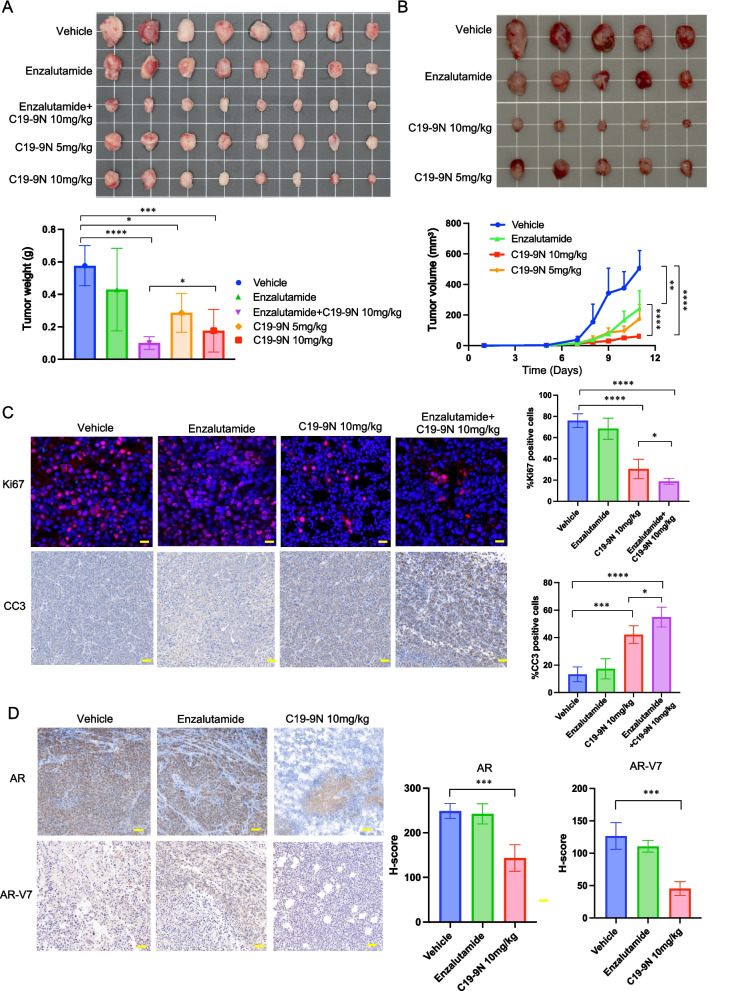
C19-9N exhibits in vivo efficacy in enzalutamide-resistant CRPC models. **A** C19-9N suppresses TRAMP-C1 xenograft growth. Top: Representative images of TRAMP-C1 tumors harvested from mice following treatment with vehicle, enzalutamide monotherapy (10 mg/kg), C19-9N at two different doses (5 and 10 mg/kg), or combination therapy (enzalutamide 10 mg/kg plus C19-9N 10 mg/kg). All treatments were administered orally once daily. Bottom: Quantitative analysis of tumor weight. **B** C19-9N inhibits 22RV1 xenograft progression. Top: Representative images of 22RV1 tumors from mice treated with vehicle, enzalutamide (10 mg/kg), C19-9N (5 mg/kg and 10 mg/kg) by orally administration twice daily. Bottom: Tumor volume growth curve over 12 days. **C** C19-9N reduces Ki-67 (proliferation marker) expression and increases Cleaved Caspase-3 (CC3, apoptosis marker) expression. Top Left: Representative IF staining of Ki-67 in TRAMP-C1 tumor sections. Top Right: Quantitative analysis of Ki-67-positive cells. Bottom Left: Representative IHC staining of CC3 in TRAMP-C1 tumor sections. Bottom Right: Quantitative analysis of CC3-positive cells. Scale bar = 50 μm. **D** C19-9N downregulates AR and AR-V7 in 22RV1 tumors. Left: Representative IHC staining of AR (Top) and AR-V7 (Bottom) in 22RV1 tumor sections (Vehicle/Enzalutamide/C19-9N groups). Scale bar = 50 μm. Right: Quantitative analysis of AR and AR-V7-positive cells. Data are expressed as mean ± SD, *P-value < 0.05, **P-value < 0.01, ***P-value < 0.001

We evaluated the in vivo efficacy of C19-9N in two preclinical models of castration-resistant prostate cancer (CRPC): an allogeneic TRAMP-C1 xenograft model (castrated mice) and a 22RV1 xenograft model (Fig. 5A-B). Via structural optimization and solvent formulation improvements, oral bioavailability of C19-9N was elevated to 43.3% (vs 15.8% the parent compound C19—9, which required injectable administration; Figure S6A), enabling oral dosing for subsequent efficacy assessments.

In castrated mice bearing TRAMP-C1 xenografts (a model of enzalutamide-resistant CRPC, used to validate in vitro synergy findings), oral C19-9N (5 or 10 mg/kg, q.d.) dose-dependently suppressed tumor growth, whereas enzalutamide (10 mg/kg, q.d.) exhibited minimal activity. Critically, none of the treatment regimens (C19-9N monotherapy, C19-9N combined with enzalutamide, or enzalutamide alone) altered animal body weight (Figure S6B), confirming favorable in vivo tolerability. As shown in Figure S6C, tumor volume measurements over 10 days confirmed that while the Enzalutamide group showed rapid progression similar to the vehicle control, treatment with C19-9N (10 mg/kg) significantly delayed tumor growth. Furthermore, the combination of Enzalutamide and C19-9N (10 mg/kg) exerted the most potent inhibitory effect, maintaining the lowest tumor volume throughout the study. Specifically, C19-9N (10 mg/kg) reduced tumor weight by 70.02% (vs. 28.81% with enzalutamide alone), and its combination with enzalutamide achieved 85.20% reduction in tumor weight (Fig. 5A). Immunohistochemical (IHC) staining further validated C19-9N’s anti-proliferative activity via significant downregulation of the proliferation marker Ki-67, and the expression of cleaved caspase-3 (CC3) was significantly upregulated, particularly in the C19-9N and combination groups, indicating the induction of apoptosis (Fig. 5C).

To rigorously evaluate the single-agent potency of C19-9N against advanced, treatment-resistant disease, we employed the AR-V7-positive 22RV1 xenograft model. Based on preliminary pharmacokinetic data (Figure S6A), a twice-daily dosing regimen was implemented for C19-9N to maximize drug exposure and assess its optimized monotherapy potential. In the 22RV1 xenograft model (AR-V7-positive CRPC), oral C19-9N (5 or 10 mg/kg) dose-dependently inhibited tumor volume progression, with inhibition rates of 64.23% and 90.58%, respectively, outperforming enzalutamide (10 mg/kg, 54.23% inhibition; Fig. 5B). No significant changes in body weight were observed in any of the treatment groups (Figure S6C). Consistent with the tumor volume data, the final tumor weights at the study endpoint further confirmed the superior efficacy of C19-9N. As shown in Figure S6D, treatment with C19-9N (10 mg/kg) resulted in a dramatic reduction in tumor mass compared to the vehicle group. Notably, the inhibitory effect of C19-9N (10 mg/kg) on tumor weight was significantly more pronounced than that of Enzalutamide, which showed only a moderate reduction. This study provides a critical benchmark for the standalone activity of C19-9N. Consistent with the findings in the TRAMP-C1 model, IHC analysis of 22RV1 xenografts revealed that C19-9N treatment significantly inhibited tumor cell proliferation, as evidenced by a marked reduction in Ki-67 positive cells, and concurrently induced apoptosis, indicated by a significant increase in CC3 positive cells (Figure S6F-G). Notably, the pro-apoptotic effect of C19-9N was more potent than that of enzalutamide. This superior efficacy is likely attributable to the C19-9N's ability to significantly downregulate the expression of both full-length AR and the splice variant AR-V7, a key driver of enzalutamide resistance (Fig. 5D).

Preliminary safety evaluations (acute and long-term toxicity studies) further supported the translational potential of C19-9N: no significant body weight changes were observed, and histopathological assessment of major organs (heart, liver, lung, kidney, prostate) revealed no overt treatment-related toxicity (Figure S7A-E). Collectively, C19-9N overcomes enzalutamide resistance and exhibits favorable safety and pharmacokinetic (PK) profile, providing a compelling preclinical rationale for the clinical development of C19-9N as a novel therapeutic strategy for enzalutamide-resistant PCa.

### C19-9N suppresses development of neuroendocrine and bone metastatic progression

**Fig. 6:**
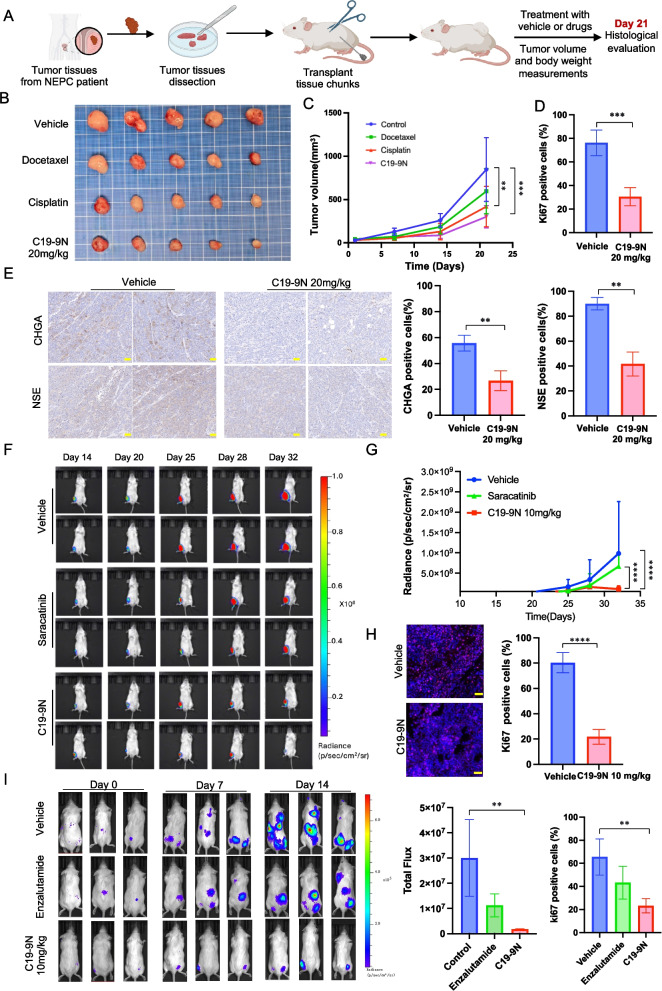
C19-9N exerts anti-tumor activity in NEPC PDX and bone metastatic CRPC models. **A** Experimental workflow for NEPC PDX model. Schematic of NEPC patient-derived xenograft (PDX) establishment. **B**-**C** C19-9N suppresses NEPC PDX growth. Representative images of NEPC PDX tumors from mice treated with vehicle, docetaxel, cisplatin, or C19-9N (20 mg/kg). **C **: Tumor volume growth curve over 25 days. **D** C19-9N reduces Ki-67 (proliferation marker) expression in NEPC PDX tumor tissues. **E** C19-9N downregulates neuroendocrine markers in NEPC PDX. Left: Representative IHC staining of neuroendocrine markers (CHGA, NSE) in vehicle vs. C19-9N-treated NEPC PDX tumors. Scale bar = 50 μm. Right: Quantitative analysis of CHGA/NSE-positive cells. **F**-**G** C19-9N’s efficacy against bone metastatic CRPC using a PC3 xenograft model. F: Longitudinal bioluminescent imaging (BLI) of bone metastases in mice treated with vehicle, saracatinib, or C19-9N (10 mg/kg). G: BLI signal intensity (tumor burden) over 32 days. **H** C19-9N reduces proliferation in bone metastatic tumors by quantitative analysis of Ki-67-positive cells. Scale bar = 50 μm. **I** C19-9N overcomes enzalutamide resistance in immune-competent bone metastatic model. Longitudinal BLI of MycCaP-Bo bone metastases in mice treated with vehicle, enzalutamide, or C19-9N (10 mg/kg) (Days 9–14). Quantitative analysis of the percentage of Ki-67 positive cells bone metastatic tumor tissues. Data are expressed as mean ± SD, *P-value < 0.05, **P-value < 0.01, ***P-value < 0.001

NEPC and bone metastatic PCa are high-grade malignancies characterized by aggressive clinical behavior, poor prognosis, and extremely limited therapeutic options [40]. Notably, integrin-mediated cell surface interactions are significantly enriched in NEPC compared to PCa [41]—supporting integrin targeting as a rational therapeutic strategy for this subtype. Given the high αv integrin expression and AR-negative status of NEPC, we established a corresponding PDX model to assess the therapeutic activity of C19-9N (Fig. 6A and Figure S7F). NEPC typically exhibits intrinsic resistance to taxane-based therapies, with platinum-based regimens being the current standard of care; however, platinum agents are associated with substantial systemic toxicity and have failed to meaningfully improve overall survival in NEPC patients. As shown in Fig. 6B-C and Figure S7G, docetaxel treatment induced only a mild reduction in tumor burden, while cisplatin exerted a more pronounced but still limited suppressive effect on tumor growth. Notably, C19-9N (20 mg/kg) achieved striking tumor growth attenuation, with tumor volumes significantly smaller than those in the docetaxel, cisplatin, and vehicle groups, and treatment with C19-9N significantly suppressed tumor cell proliferation, as evidenced by a marked reduction in the percentage of Ki67-positive cells, highlighting its superior in vivo anti-tumor efficacy in NEPC(Fig. 6D). Given that NEPC is defined by neuroendocrine differentiation and expression of markers such as CHGA and NSE, IHC analysis confirmed that C19-9N significantly downregulated the expression of these neuroendocrine markers (Fig. 6E).

We next evaluated the efficacy of C19-9N against bone metastatic CRPC using a PC3-based xenograft model. SRC kinase inhibitors have emerging as promising anti-osteolytic agents in both preclinical and clinical studies, with saracatinib advancing in clinical trail for solid tumors [42]. Quantitative bioluminescent imaging (BLI) analysis over the course of the study revealed that C19-9N (10 mg/kg) administration resulted in a dramatic and sustained suppression of bone metastatic burden, effectively halting disease progression. Compared to the vehicle control, C19-9N not only significantly inhibited tumor growth but also outperformed the positive control saracatinib (25 mg/kg), which showed only a modest reduction in signal intensity. Strikingly, while the tumor burden in control groups continued to escalate, the C19-9N-treated cohort exhibited minimal residual radiance, suggesting a potential for lesion regression (Fig. 6F-G).Consistent with its anti-proliferative activity in other models, IHC analysis of the proliferation marker Ki-67 confirmed a significant reduction in the proliferative index of C19-9N-treated tumors (Fig. 6H). In parallel, the CC3 was notably increased, demonstrating that C19-9N also promotes apoptosis (Figure S8).

The capacity of C19-9N to overcome enzalutamide resistance was investigated using the MycCaP-Bo model—an immunocompetent in vivo system for metastatic PCa [25]. This bone-tropic subline was generated via three rounds of in vivo selection for bone-homing cells, following intracardiac (IC) inoculation of MycCaP cells—an androgen-dependent murine prostate cancer cell line derived from the FVB/N background—into syngeneic mice. For longitudinal in vivo tracking, MycCaP-Bo cells were stably transduced with a lentiviral vector expressing firefly luciferase (Fluc), enabling non-invasive detection and quantitative monitoring of bone metastases via BLI. As quantified by BLI analysis (Fig. 6I), MycCaP-Bo-derived bone lesions developed resistance to enzalutamide after 14 days of treatment—consistent with our prior findings [25]. In striking contrast, C19-9N administration exerted a significant inhibitory effect on the progression of established bone lesions, effectively abrogating enzalutamide-resistant tumor outgrowth. IHC of harvested tumor tissues further demonstrated that C19-9N treatment led to a marked reduction in the proliferation marker Ki-67 and a concurrent increase in the apoptotic marker CC3 (Fig. 6I, Figure S8), thereby recapitulating the dual anti-proliferative and pro-apoptotic effects observed in vitro.

### C19-9N modulates tumor immune microenvironment by targeting TAMs and CD47 in resistant bone metastases

**Fig. 7:**
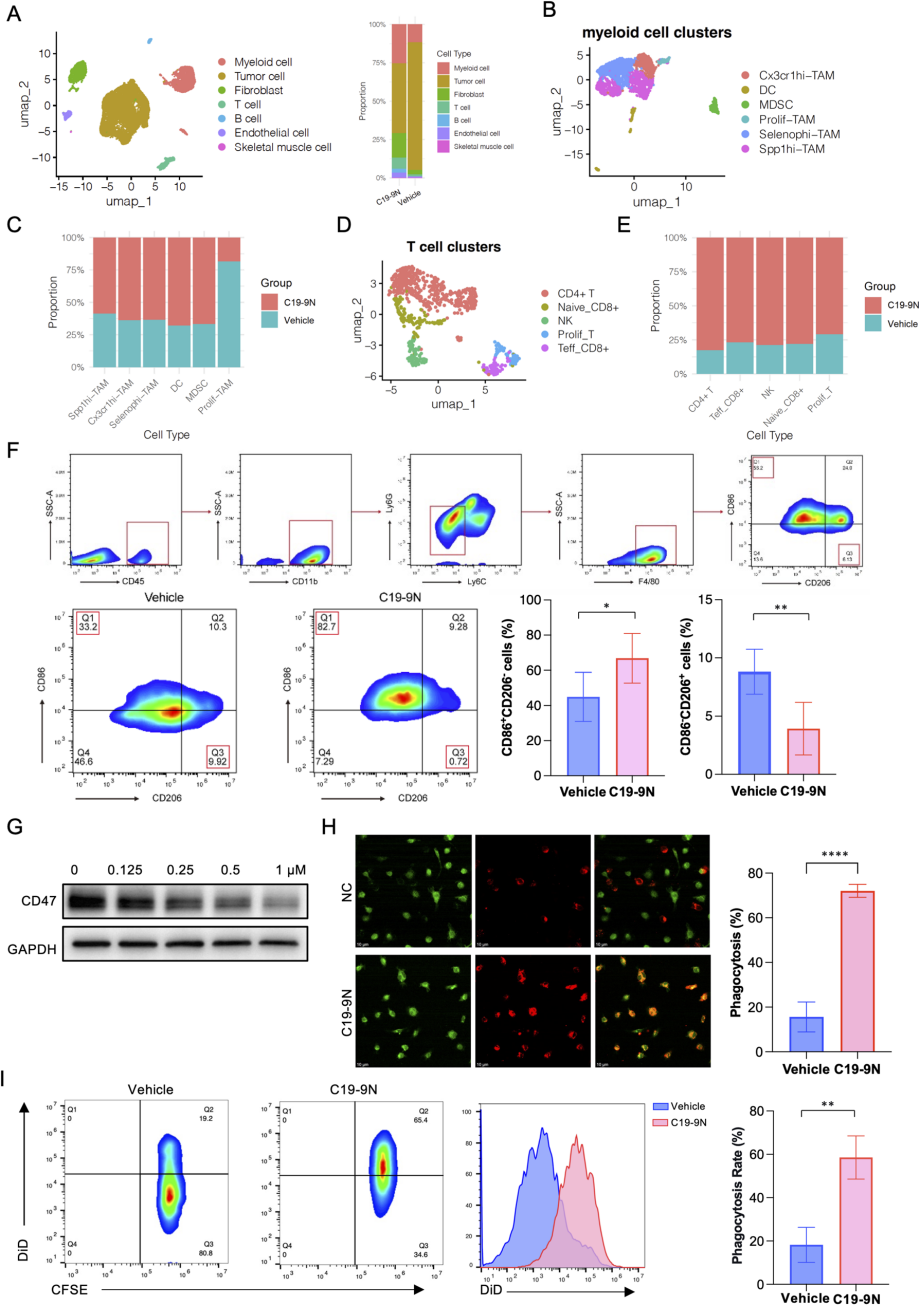
C19-9N remodels TME immune landscape via TAM polarization and CD47-mediated phagocytosis enhancement. **A** scRNA-seq landscape of tumor-infiltrating cells. Left: UMAP plot of major cell types in MycCaP-Bo bone lesions. Right: Bar plot showing cell type proportion changes (C19-9N vs. vehicle). **B** Myeloid cell subset clustering. UMAP plot of myeloid clusters in the TME. **C** Myeloid subset proportion changes. Bar plot showing relative proportions of myeloid subsets in C19-9N vs. vehicle groups. **D** T cell subset clustering. UMAP plot of T cell clusters in the TME. **E** T cell subset proportion changes. Bar plot showing relative proportions of T cell subsets in C19-9N vs. vehicle groups. **F** Flow cytometry gating strategy and TAM polarization analysis. Top: Sequential gating strategy for identifying TAMs (from total live cells to CD45⁺CD11b⁺F4/80⁺ subsets), followed by CD86/CD206 staining. Bottom: Flow plots (vehicle vs. C19-9N) and quantitative analysis of CD86⁺CD206⁻ and CD86⁻CD206⁺ TAM proportions. **G** C19-9N dose-dependently downregulates CD47. **H-I** C19-9N enhances macrophage phagocytosis. H: Confocal microscopy of CFSE-labeled tumor cells (green) and DID-labeled macrophages (red) (vehicle vs. C19-9N; Scale bar = 10 μm). Right: Quantitative phagocytosis rate. Data are expressed as mean ± SD, *P-value < 0.05, **P-value <0.01, ***P-value < 0.001

Previously, we found that macrophages are particularly important for the development of anti-androgen resistance in bone metastatic PCa. Macrophages and CD47-driven immune escape are key mediators in PCa tumor microenvironment (TME) [41]. To define the impact of C19-9N on TME immune cell dynamics, we performed single-cell RNA sequencing (scRNA-seq) on MycCaP-Bo bone lesions (C19-9N vs. vehicle treatment). scRNA-seq of tumor-infiltrating cells confirmed C19-9N remodeled myeloid and T cell populations (Fig. 7A, Figure S9A-B). Macrophages were identified by showing high expression levels of CD68. The unbiased analysis identified four TAMs populations with distinct gene expression profiles, and among them, Selenophi-TAM accounted for the largest proportion (Fig. 7B; Figure S9C). Selenophi TAM macrophages are a specific subset in the TME with pro-inflammatory functions, which also confirmed by irGSEA analysis (Figure S9D) and immunosuppression score (Figure S9E). Recent studies had demonstrated that the TNF signaling pathway and NF-κB pathway were significantly enriched in Selenop⁺ macrophages, which could secrete pro-inflammatory cytokines such as TNF-α and IL-1β to recruit and activate CD8⁺ T cells [43]. C19-9N significantly reduced the proportion of prolifhi-TAM (Fig. 7C). The result of irGSEA analysis suggested prolifhi-TAM were mainly anti-inflammatory phenotype, because their proliferation was dependent on anti-inflammatory signals within the TME (e.g., IL-4 and CSF-1), and they concurrently inhibited CD8⁺ T cell activity via secreting IL-10 and TGF-β, thereby promoting tumor progression [43]. Next, we evaluated the effect of C19-9N on the composition of T-cell infiltrates. As shown in Fig. 7D-E and Figure S9F, a significant increase in the proportion of CD8^+^T and NK cells was observed following treatment with C19-9N. IHC result also suggested C19-9N could increase the abundance of CD8^+^Ki67^+^ T cell population (Figure S9G).

To confirm TAMs polarization reprogramming in enzalutamide-resistant bone metastases, we isolated TAMs via fluorescence-activated cell sorting (FACS) from 14-day treated MycCaP-Bo lesions (the gold standard for myeloid subset purification). Flow cytometry showed C19-9N increased CD86^+^CD206^−^ pro-inflammatory TAMs (P < 0.05) and reduced CD86^−^CD206^+^ immune-suppressive TAMs (Fig. 7F), which drives anti-androgen resistance in bone lesions [44]. This was recapitulated in tumor supernatant-induced BMDM polarization assays. C19-9N caused an extremely significant reduction in CD206^+^ TAMs (Figure S9H). These findings demonstrate that C19-9N shifts TAM polarization toward a pro-inflammatory phenotype, suggesting a potential mechanism by which C19-9N mitigates the progression of enzalutamide-resistant bone metastases.

scRNA-seq revealed that C19-9N treatment led to a significant reduction of CD47 expression within the tumor (Figure S9I). CD47 is a ubiquitously expressed cell surface integrin-associated protein, and also known as the “don’t eat me” signal, is elevated in advanced prostate cancer and is modulated by inflammatory changes in the tumor microenvironment [45]. Western blot analysis demonstrated C19-9N elicited dose-dependent suppression of CD47 expression (Fig. 7G), highlighting its potential to abrogate CD47-mediated immune escape and enhance macrophage-driven phagocytosis of tumor cells, which further validated in Fig. 7H-I. Confocal microscopy revealed that C19-9N treatment markedly increased colocalization between CFSE-labeled tumor cells (green) and DiD-labeled macrophages (red), consistent with enhanced phagocytic uptake. Flow cytometry-based phagocytosis assays further corroborated these observations (Fig. 7I), C19-9N doubled phagocytosis of CFSE^+^ tumor cells by DiD^+^ macrophages, with expanded signal overlap in the overlay histogram. Collectively, these results establish two immune-mediated mechanisms of C19-9N: (1) remodeling TAMs polarization to reduce anti-inflammatory subsets and enhance cytotoxic lymphocyte infiltration; (2) downregulating CD47 to disrupt “don’t eat me” signaling and promote macrophage phagocytosis, which expanding its anti-tumor activity to TME immune modulation.

## Discussion

**Fig. 8:**
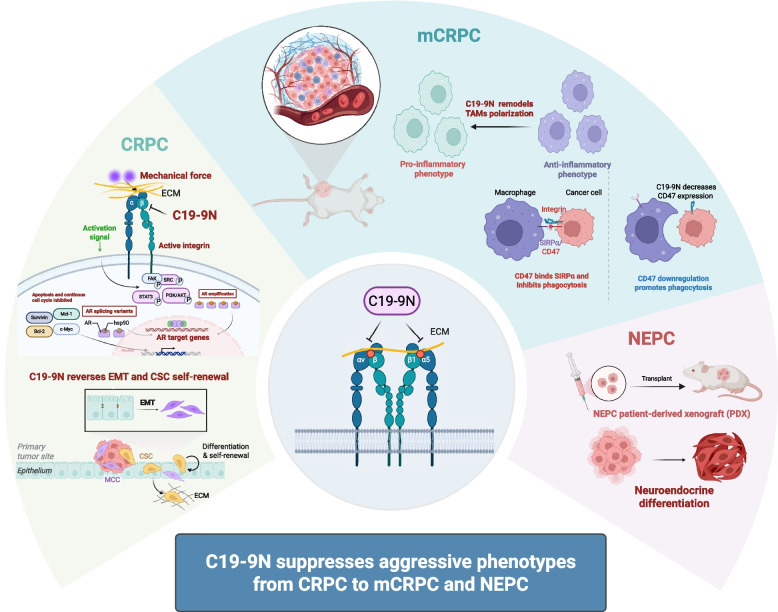
Schematic illustration of C19-9N-mediated suppression of aggressive phenotypes across castration-resistant (CRPC), metastatic castration-resistant (mCRPC), and neuroendocrine (NEPC) prostate cancer subtypes

Advanced PCa, particularly CRPC, NEPC, and bone metastatic CRPC, remains a major clinical challenge due to the high incidence of therapy resistance and limited effective therapeutic options. Integrin-ECM crosstalk is a central driver of CAMDR, CSC maintenance, EMT and immune escape—key hallmarks of aggressive PCa progression [11]. In this study, we rationally designed C19-9N, a novel pan-αv and α5β1 integrin antagonist, and systematically validated its preclinical efficacy and mechanisms of action in overcoming therapy resistance and suppressing aggressive PCa phenotypes. Our findings established C19-9N as a promising multi-targeted therapeutic agent with unique advantages in addressing unmet clinical needs in advanced PCa (Fig. 8).

Previous studies, including our own, have primarily focused on individual integrin subtypes, which fails to account for the functional redundancy of integrins in PCa progression. A central challenge in targeting integrin signaling lies in the compensatory crosstalk between integrin subtypes: inhibition of a single integrin (e.g., αvβ3) often triggers upregulation or hyperactivation of alternative integrins (e.g., αvβ5, α5β1) that reinforce cell adhesion, survival, and metastasis [46–48]. This subtype redundancy has plagued single-target integrin inhibitors in clinical development, limiting their efficacy against heterogeneous CRPC. The rational design of C19-9N addresses this critical limitation of existing integrin-targeting agents. C19-9N’s design as a pan-αv/α5β1 integrin antagonist directly circumvents potential compensatory resistance mediated by integrin subtype switching. Notably, C19-9N is an orally non-RGD small molecule that binds integrins independently of Mn^2^⁺, distinguishing it from RGD-based ligands that rely on divalent cation-induced conformational changes (Fig. 1, Figure S2). Mechanistically, our findings delineated multifaceted roles for C19-9N, whereby it concomitantly targets interconnected oncogenic and microenvironmental pathways to surmount enzalutamide resistance and suppress aggressive PCa progression.

C19-9N exerts broad anti-tumor effects across diverse PCa subtypes, a critical feature given the molecular heterogeneity of advanced PCa. It potently inhibits proliferation of AR-positive CRPC (22RV1), AR-negative mCRPC (PC3), and NEPC (NCI-H660) cells, indicating efficacy regardless of AR status or neuroendocrine differentiation (Fig. 2). In preclinical models, C19-9N overcomes enzalutamide resistance in CRPC xenografts, suppresses bone metastatic progression, and exhibits superior efficacy in NEPC PDX compared to platinum/taxane regimens(Fig. 5). Notably, C19-9N shows favorable oral bioavailability (43.3%) and a satisfactory safety profile (Figure S6).

C19-9N enhances enzalutamide sensitivity by promoting apoptosis, dysregulating cell cycle progression, and modulating Survivin, c-Myc, Mcl-1 and Bcl-2 (Fig. 2). In addition, C19-9N inhibits SRC, PI3K/AKT, and STAT3 phosphorylation, suppressing AR alternative splicing and AR-V7—key enzalutamide resistance drivers (Fig. 4). IL6/STAT3 signaling is associated with endocrine therapy resistance in PCa [39]. The IL-6/STAT3/AR axis is a well-characterized resistance circuit where inflammatory cytokines (e.g., IL-6) or constitutive oncogenic signaling activate STAT3, which in turn co-opts AR signaling, promoting its transcriptional activity, facilitating the expression of splice variants like AR-V7, and ultimately driving tumor survival and progression [39]. Therefore, targeting the IL-6/STAT3/AR signaling axis represents a pivotal therapeutic strategy to overcome enzalutamide resistance in PCa. C19-9N’s pan-integrin inhibition acts as an upstream master switch, intercepting the key signaling cascades that drive therapy resistance and thereby preventing compensatory pathway activation through alternative integrin heterodimers. This direct engagement results in a potent, dose-dependent downregulation of the critical resistance driver AR-V7 at both the mRNA and protein levels. The specificity of this action is underscored by the fact that genetic silencing of ITGAV recapitulates the same suppressive effect, confirming αv integrin as a singularly sufficient target to disrupt AR-V7 signaling. We further substantiated the robustness of this mechanism by demonstrating consistent downregulation of AR-FL and AR-V7 in the C4—2B enzalutamide-resistant model (Figure S5), confirming broad applicability beyond a single cellular context. The functional consequence of this inhibition is a profound disruption of AR nuclear trafficking, leading to markedly suppressed AR transcriptional activity and consequent downregulation of canonical target genes such as TMPRSS2 and KLK2.

The mechanistic rationale for the observed synergy with enzalutamide lies in C19-9N’s ability to recalibrate, rather than obliterate, the hyperactive AR signaling landscape characteristic of resistant disease. By partially but significantly reducing the overwhelming burden of AR-FL and AR-V7, C19-9N lowers the threshold for effective receptor antagonism. It simultaneously diminishes the tumor's reliance on compensatory non-canonical survival pathways, effectively funneling its dependency back towards the AR-FL axis. This renders the cancer cell newly vulnerable to the direct receptor blockade exerted by enzalutamide. This multi-layered regulatory strategy, simultaneously targeting the expression, localization, and transcriptional competence of the AR axis, represents a paradigm distinct from existing agents that primarily focus on ligand competition or receptor degradation. By resetting the pathological AR signaling network to a drug-sensitive state, C19-9N provides a novel and compelling therapeutic strategy to overcome AR-V7-mediated resistance to next-generation antiandrogens.

C19-9N disrupts ECM-integrin crosstalk, abrogates mechanotransduction signaling, and reverses mechanoregulated stemness-related gene expression and EMT, critical drivers of metastasis and therapy resistance. ECM stiffness, mimicked by Matrigel, upregulates αvβ3 and α5β1 expression and induces CSC-like phenotypes in PCa cells—consistent with the role of integrins in transducing biomechanical signals. C19-9N inhibits sphere formation and downregulates stemness-associated genes (SOX2, CD44 and ALDH1A1) (Fig. 3), directly suppressing CSC self-renewal. Concurrently, it reverses EMT by increasing E-cadherin and decreasing vimentin expression, explaining its inhibitory effects on cell migration and invasion. These findings connect αvβ3/α5β1 inhibition with the suppression of two key, interrelated metastatic phenotypes in PCa, thereby expanding the therapeutic relevance of C19-9N to bone metastasis.

Beyond direct anti-tumor effects, C19-9N also exerts prominent immunomodulatory activity in bone metastatic PCa, including reprogramming TAMs polarization, downregulating CD47, and enhancing macrophage phagocytosis. Mechanistically, integrin subtype redundancy is a key driver of immune-suppressive TME remodeling and bone tropism in advanced prostate cancer [49]. Bone metastasis represents a major source of morbidity and mortality in PCa, and anti-androgen resistance in osseous lesions is frequently driven by TME-dependent mechanisms—posing a significant clinical challenge [50]. Our preclinical data demonstrate that C19-9N effectively halts bone metastatic progression in a PC3 xenograft model, outperforming saracatinib, a clinically relevant SRC kinase inhibitor under investigation for anti-osteolytic therapy. More importantly, in the immune-competent MycCaP-Bo model of enzalutamide-resistant bone metastasis, which is an established preclinical model that recapitulates the clinical setting of therapy-resistant osseous disease, C19-9N abrogates tumor outgrowth, validating its efficacy in a translationally relevant context. This bone-metastasis-suppressive efficacy is, in part, attributed to C19-9N-mediated remodeling of the TME landscape. Integrated scRNA-seq and flow cytometry data showed that C19-9N reshapes TAM subsets, reducing immunosuppressive CD86^−^CD206^+^ cells while increasing pro-inflammatory CD86^+^CD206^−^ cells, alongside enhanced infiltration of CD8⁺ T cells. Despite the abundant expression of both αvβ3 and CD47 across a broad spectrum of malignancies, monotherapies targeting either αvβ3 or CD47 individually have been shown to yield suboptimal therapeutic efficacy for cancer treatment due to costabilization of CD47 and integrin αvβ3 on cancer cell surfaces [51]. Herein, we found C19-9N downregulates CD47, which effectively disrupts this costabilization, which enhances macrophage phagocytosis and thereby promoting macrophage-dependent tumor cell clearance. This dual immunomodulatory mechanism, characterized by TAM polarization reprogramming and CD47-dependent “don’t eat me” signal ablation, represents a novel and translationally relevant dimension of C19-9N’s anti-tumor activity. This is particularly significant given the limited efficacy of immune checkpoint inhibitors in PCa, highlighting C19-9N’s potential to address unmet needs in TME-targeted therapy for advanced PCa. The translational potential of C19-9N is further supported by its favorable pharmacokinetic and safety profiles.

In conclusion, our study identifies C19-9N as a first-in-class, orally bioavailable pan-αv/α5β1 integrin antagonist that overcomes enzalutamide resistance and suppresses aggressive phenotypes, including CRPC, bone metastasis and NEPC. Its unique pan-integrin targeting strategy addresses the fundamental limitation of single-subtype integrin inhibitors (e.g., cilengitide) by eliminating tumor cell "escape routes" via compensatory integrin activation, ensuring robust and durable anti-tumor efficacy. Given its versatile mechanism, favorable pharmacokinetic profile, and ability to overcome both intrinsic and acquired resistance, C19-9N represents a promising candidate for clinical development in patients with advanced PCa, particularly those with enzalutamide-resistant, AR-V7-positive, or NEPC subtypes.

Despite these promising findings, this study has limitations. First, while we validated C19-9N’s efficacy in multiple preclinical models, including PDX and immune-competent models, clinical translation will require further evaluation in phase I/II trials to determine optimal dosing, safety, and efficacy in patients with advanced PCa. Second, while our mechanistic studies identify key pathways (SRC/AKT/STAT3, AR-V7, TAM polarization, CD47), the precise downstream signaling networks mediating C19-9N’s effects remain to be fully elucidated. For example, the interplay between integrin inhibition and CD47 downregulation warrants further investigation. Third, while we focused on αv and α5β1 integrins, C19-9N also binds αvβ6 and αvβ8, which may contribute to its anti-tumor activity in other solid tumors. Finally, combination studies with other therapeutic modalities (e.g., immune checkpoint inhibitors, chemotherapy) in advanced PCa models will help define C19-9N’s role in multi-agent regimens.

## Materials and methods

### Patients and samples

This study utilized formalin-fixed, paraffin-embedded (FFPE) tissue specimens obtained from a cohort of twenty prostate cancer patients who underwent surgical resection at Peking University First Hospital between 2021 and 2024 and received ethical approval from Peking University First Hospital (2023R052 and 2023R035). The cohort was comprised of two distinct clinical groups. The first group (*n* = 10) consisted of primary prostate adenocarcinoma samples from treatment-naïve patients at the time of radical prostatectomy. The second group (*n* = 10) included metastatic castration-resistant prostate cancer (mCRPC) samples, which were collected from patients who subsequently developed tumor recurrence, met the clinical criteria for CRPC, and had confirmed bone metastases.

### Homology modeling and molecular docking in silico

Template crystal structure was identified through BLAST and downloaded from RCSB Protein Data Bank (PDB ID: 1L5G). Models are computed by the SWISS-MODEL server1 homology modelling pipeline which relies on ProMod3, an in-house comparative modelling engine based on OpenStructure. ProMod3 extracts initial structural information from the template structure. Insertions and deletions, as defined by the sequence alignment, are resolved by first searching for viable candidates in a structural database. Final candidates are then selected using statistical potentials of mean force scoring methods. If no candidates can be found, a conformational space search is performed using Monte Carlo techniques. Non-conserved side chains are modelled using an in-house backbone-dependent rotamer library. The optimal configuration of rotamers is estimated using the graph-based TreePack algorithm by minimizing the SCWRL4 energy function. As a final step, small structural distortions, unfavorable interactions or clashes introduced during the modelling process are resolved by energy minimization. ProMod3 uses the OpenMM library to perform the computations and the CHARMM22/CMAP force field for parameterization.

The crystal structures (PDB ID: 4UM9 for αvβ6, 3VI4 for α5β1, 1L5G for αvβ3) were obtained from RCSB Protein Data Bank (PDB) (https://www.rcsb.org). The ligands were prepared by using energy minimization, and the protein structure was prepared by using QuickPrep Wizard (MOE 2022). The oriented compounds then saved as a new data base by MDB format. Thirty poses were generated for each docking process, which were regulated by the London dG-scoring function, and adjusted twice using the triangle Matcher method. The best poses obtained by comparing the interaction with key residues were output among the docking poses. The results are presented using Pymol software.

### Surface Plasmon Resonance (SPR) and MicroScale Thermophoresis (MST) Assay

To inspect the potential hits with binding affinities to integrin family, SPR technology-based BIAcore 8 K (GE Healthcare, USA) was applied according to the manufacturer’s instruction. Briefly, integrin protein αvβ3, αvβ5, αvβ6, αvβ8, α5β1 (ACROBiosytsems, China) was immobilized to CM5 chip (Cytiva, USA) by the amine-coupling method. The bound ligands were diluted in PBS-P buffer (10 mM PBS, 150 mM NaCl, 0.05% pH7.4, 0.05% v/v Surfactant P20) and injected into the flow cells at a flow rate of 30 μL/min. The sensor surfaces were regenerated with a short pulse of 50% DMSO. All SPR measurements were performed at 25 °C. Data were analyzed by the Biacore 8 K Evaluation Software (GE Healthcare, USA) for ligand-integrin binding affinity.

MST experiments were performed on a Monolith NT.115pico (NanoTemper Technologies GmbH, Germany). Integrin αvβ3 protein (200 nM) was labeled with Monolith NT™ Protein Labeling Kit Red-NHS in HBS-P buffer (0.01 M HEPES pH7.4, 0.15 M NaCl, 0.05% v/v Surfactant P20). The human Fibronectin was diluted in HBS-P buffer with MnCl_2_, and the C19-9N was diluted in HBS-P buffer with or without MnCl_2_. To study the effect of C19-9N on the binding ability of Fibronectin, αvβ3 incubated with C19-9N for 2 h was pretreated before MST test. 10 µL diluted ligand at various concentrations were mixed with 10 µL labeled protein, and then were loaded to standard capillaries. Automated MST measurement was carried out with Medium MST power. KD value was calculated based on a dose–response curve using MO. Affinity Analysis software (Nano Temper Technologies GmbH, Germany).

### Cell culture

A panel of human prostate cancer cell lines, including PC3, 22RV1, NCI-H660 and LNCaP, was obtained from the Cell Bank of the Chinese Academy of Sciences (Shanghai, China), and C4—2B was obtained from Zhong Qiao Xin Zhou Biotechnology Co.,Ltd.(Shanghai, China). The MycCaP-Bo, a murine prostate cancer cell line with high bone metastatic potential, was kindly provided by Professor Binzhi Qian from the University of Edinburgh. All cell lines were maintained in their respective recommended media (Gibco, USA), supplemented with 10% fetal bovine serum (FBS; Sigma-Aldrich, F8318, USA) and 1% penicillin–streptomycin, and incubated at 37 °C in a humidified atmosphere containing 5% CO₂. The docetaxel-resistant DU145 was kindly provided by the Institute of Urology of Peking University. The docetaxel-resistant DU145 subline (DU145-DR) was established through chronic, escalating exposure to docetaxel to recapitulate the adaptive pressures of clinical chemoresistance. Briefly, parental DU145 cells were subjected to sequential 24-h pulses of docetaxel, beginning at 1 nM, followed by recovery in standard growth medium (RPMI-1640 supplemented with 10% FBS). This pulse-recovery cycle was repeated three times at each concentration before escalating to the next dose. Through iterative application of this regimen across a defined concentration gradient (2, 4, 6, 8, 10, 15, 20, 25, 30, 35, 40, 45, and 50 nM), a stably resistant population capable of sustained proliferation in 50 nM docetaxel(TargetMol, T1034, USA) was selected and designated DU145-DR. To maintain the acquired resistant phenotype, DU145-DR cells are routinely cultured in complete medium supplemented with 10 nM docetaxel.

### Cellular thermal shift assay (CETSA)

22RV1 cells were treated with control (DMSO) or C19-9N (0.5 μM) and incubated at 37˚C for 3 h. Cells were washed once and divided into aliquots with 0.5 × 10^6^ cells each. Samples were heated pairwise (control and C19-9N-treated samples) for 4 min at 40–64˚C with 3˚C increments between pairs. After heating, cells were kept at room temperature for 3 min before transferring to ice. Cells were collected and lysed in NP-40 lysis buffer (50 mM Tris–HCl pH 7.4, 10% glycerol, 50 mM NaCl, 0.5% sodium deoxycholate, 1% NP-40, 20 mM NaF) supplemented with 1 mM PMSF and protease inhibitor cocktail (MedChemExpress, China), subjected to two freeze–thaw cycles using liquid nitrogen and a 37 °C water bath. The lysates were centrifuged at 20,000 g for 20 min at 4 °C, and then the supernatants were subsequently analyzed by SDS-PAGE and Western blotting using integrin αv, integrin α5 and GAPDH antibodies. Protein levels were quantified with G:Box chemi XX9 (Syngene, UK) and normalized to 40˚C samples for both treatments.

### Isolation and differentiation of bone marrow-derived macrophages (BMDMs)

Bone marrow cells were isolated from the femurs and tibias of euthanized FVB mice. Following sterilization in 75% ethanol, the bones were flushed with RPMI-1640 (Gbico, C11875500BT, USA) using a syringe to extract the marrow. The cell suspension was filtered through a 70 µm cell strainer (Corning, 352,350, USA), centrifuged, and treated with red blood cell lysis buffer (Solarbio, R1010, China) to remove erythrocytes. The cells were then resuspended in complete RPMI-1640 containing 10% fetal bovine serum (FBS, Sigma-Aldrich, F8318, USA) and 20 ng/mL mouse M-CSF recombinant protein (Peprotech, 315—02, USA) to induce macrophage differentiation. Cells were plated and cultured at 37 °C with 5% CO₂, and the medium was partially refreshed on days 3 and 5. Fully differentiated BMDMs were obtained by day 6—7 for use in downstream assays.

### Cell viability assay

Cell viability was assessed using the Cell Counting Kit-8 (CCK-8; Dojindo, Japan). Briefly, tumor cells were seeded in 96-well plates at a density of 2 × 10^3^ cells per well in 100 µL of culture medium and allowed to adhere overnight. The following day, the cells were treated with either fresh medium (control) or various concentrations of C19-9N for 48 to 72 h. Subsequently, 10 µL of CCK-8 solution was added to each well, and the plates were incubated at 37 °C for 1 h. The absorbance at 450 nm was measured using a microplate reader. The half-maximal inhibitory concentration (IC₅₀) was calculated by fitting the dose–response data to a four-parameter logistic model using GraphPad Prism software (Version 7.0).

### Cell migration and invasion assay

Cell migration and invasion were assessed using 24-well Transwell chambers (Corning, 3422, USA). For the invasion assay, the upper chamber was pre-coated with a thin layer of matrigel (Corning, 356,234, USA) to simulate the extracellular matrix, whereas the migration assay was performed without coating. Briefly, cells pretreated with or without C19-9N were seeded into the upper chamber in serum-free medium. The lower chamber was filled with medium supplemented with 10% FBS as a chemoattractant. After incubation at 37 °C for 24 h, non-migratory/invasive cells on the upper surface of the membrane were carefully removed with cotton swabs. Cells that had traversed the membrane were fixed with 4% paraformaldehyde, stained with 0.5% crystal violet, and imaged under a microscope. The number of migrated or invaded cells was quantified by counting stained cells in three random fields per well.

### Phagocytosis assay

BMDMs were labeled with CFSE (BD Biosciences, 565,082, USA) and seeded in 24-well plates at a density of 1 × 10^5^ cells per well to serve as phagocytes. Target cells (MycCaP-Bo) were pre-treated with C19-9N or vehicle for 48 h and subsequently labeled with 5 µM DiD fluorescent dye (MedChem Express, HY-D1028, USA) for 30 min at 37 °C. These labeled target cells were then added to the BMDM monolayers at an effector-to-target ratio of 1:3. The co-culture was incubated for 2 h to allow phagocytosis to occur. Following incubation, the medium was aspirated, and the cells were washed with PBS (Gibco, C14190500BT, USA). For flow cytometric analysis, cells were detached using trypsin (Gibco, 25,200,072, USA) and analyzed using CytoFlex LX (Beckman Coulter, USA). Alternatively, for imaging, cells were fixed with 4% paraformaldehyde (PFA, Biosharp, BL539A, China) for 15 min. Fluorescent images were acquired directly using a confocal laser scanning microscope (Zeiss, Germany).

### 3D matrigel culture

DU145 and PC3 cells were mixed with pre-chilled Matrigel on ice to form a homogeneous cell–matrix suspension. Aliquots of the mixture, each containing 1 × 10^4^ cells in 50 µL of Matrigel, were plated into a 24-well plate and allowed to polymerize at 37 °C for 30 min. Subsequently, 500 µL of culture medium was carefully added to each well. The cells were maintained in this 3D culture system for 7 days. Following the incubation period, the Matrigel constructs were dissolved using Cell Recovery Solution (Corning, USA) to harvest the cells for subsequent analysis. Cellular morphology was examined and compared between conventional 2D monolayer cultures and 3D Matrigel cultures using bright-field microscopy.

### Real-time PCR

Total RNA was extracted using RNAiso Plus (Takara, 9109, Japan) and subsequently reverse transcribed into cDNA with the PrimeScript RT-PCR Master Mix kit (Takara, RR036A-1, Japan) according to the manufacturer’s instructions. Quantitative real-time PCR was carried out using TB Green Premix Ex Taq (Takara, RR820A, Japan) on a 7500 Real-Time PCR System (Applied Biosystems, USA) with a two-step amplification protocol under the following cycling conditions: initial denaturation at 95 °C for 30 s, followed by 40 cycles of 95 °C for 5 s and 60 °C for 30 s. Relative mRNA expression levels were determined using the 2^^(−△△Ct)^ method.

### Flow cytometry analysis

All single-cell suspensions, whether derived from cultured cells or dissociated tissues, were washed once with cold FACS buffer (PBS supplemented with 2% FBS). For surface staining, cells were first stained with a viability dye to exclude dead cells (7-AAD, BD Biosciences, 559,925, USA), followed by a blockade of Fc receptors to minimize non-specific antibody binding (TruStain FcX ™ PLUS anti-mouse CD16/32, Biolegend, 156,603, USA). Subsequently, the cells were incubated with fluorochrome-conjugated antibodies or their corresponding isotype controls in the dark at 4 °C for 30 min. The antibodies used included: PE/Cyanine7 anti-mouse/human CD11b (BioLegend, 101,216, USA)**,** FITC anti-mouse F4/80 (BioLegend, 123,108, USA)**,** APC/Fire™ 750 anti-mouse Ly-6G (BioLegend, 127,652, USA)**,** Brilliant Violet 785™ anti-mouse Ly-6C (BioLegend, 128,041, USA)**,** Brilliant Violet 650™ anti-mouse CD86 (BioLegend, 105,036, USA)**,** and PE anti-mouse CD206 (MMR) (BioLegend, 141,706, USA). Apoptosis was assessed using an Annexin V-FITC/Propidium Iodide (PI) apoptosis detection kit (Beyotime, C1062S, China), following the standard protocol. For cell cycle analysis, cells were harvested and fixed in 70% ice-cold ethanol overnight at 4 °C, and then washed with cold PBS and treated with RNase A (100 µg/mL) to digest RNA. Cellular DNA was then stained with propidium iodide (PI, 50 µg/mL) for 30 min at room temperature in the dark. All samples were analyzed using cytometer (Beckman Coulter, CytoFlex LX, USA), and the data acquired were processed with FlowJo software (version 10.4.0).

### IHC and multiplex immunofluorescence (mIF) staining of tissue samples

FFPE tissue Sects. (5 μm) were subjected to a standardized protocol of deparaffinization, rehydration, and heat-induced antigen retrieval using a high-pH EDTA buffer (pH 9.0), which was optimized for enhanced epitope unmasking in tissue samples. For IHC, endogenous peroxidase was blocked, and sections were incubated with primary antibodies overnight at 4 °C: anti-PSMA (Cell Signal Tech, 12815S, USA), anti-ITGA2 (Abcam, ab181548, UK), anti-ITGA5 (Abcam, ab150361, UK), anti-ITGA6 (Abcam, ab181551, UK), anti-ITGAV (Abcam, ab179475, UK), anti-Ki67 (Abcam, ab15580, UK), anti-Cleaved Caspase-3 (Cell Signal Tech, 9661S, USA), anti-Chromogranin A (Abcam, ab283265, UK), anti-ENO2 (Cell Signal Tech, 24330S, USA), Anti-Synaptophysin (Abcam, ab32127, UK) followed by detection with a polymer-HRP system and 3,3'-Diaminobenzidine (DAB) development. For multiplex IF, an iterative staining protocol was employed, involving sequential rounds of primary (anti-E-cadherin (Cell Signal Tech, 3195S, USA), anti-Vimentin (Cell Signal Tech, 5741S, USA), anti-AR (Cell Signal Tech, 5153S, USA), anti-AR-V7 (Abcam, ab198394, UK), anti-Ki67 (Abcam, ab15580, UK)) and secondary antibody incubation, each followed by a heat-mediated antibody stripping step to prevent cross-reactivity, enabling the precise labeling of multiple targets on the same section. Following staining, all slides were digitally scanned using a high-resolution slide scanner. Quantitative analysis was performed using automated image analysis software. IHC expression was quantified via the H-score system across five random fields per sample, while IF signal intensity and co-localization were analyzed using Image J, providing an objective assessment of protein expression and distribution.

### Western blotting

Total protein was extracted from the cell lines or the xenograft tumors, and examined by Western blot following our previous published protocols. In brief, cells or tumor tissue were lysed on ice with RIPA buffer (Solarbio, R0010, China) and centrifuged at 4˚C. After adding loading buffer (Epizyme, LT101, China), the lysis mixture was heated at 100 °C for 10 min. Equivalent amounts of protein were separated with 10—12% SDS-PAGE and transferred to PVDF membranes (Millipore, USA). Membranes were blocked with 5% skim milk powder (Epizyme, PS112L, China) in TBST and then incubated with primary antibodies overnight at 4 °C: anti-Mcl-1 (Cell Signal Tech, 4572S, USA), anti BCL-2 (Cell Signal Tech, 15071 T, USA), anti-C-myc (Cell Signal Tech, 18583 T, USA), anti-Survivin (Cell Signal Tech, 2808 T, USA), anti-AR (Cell Signal Tech, 5153S, USA), anti-AR-V7 (Abcam, ab198394, UK), anti-ITGAV (Abcam, ab179475, UK), anti-SRC (Abcam, ab133283, UK), anti-phospho-SRC (Abcam, ab40660, UK), anti-AKT (Cell Signal Tech, 4691 T, USA), anti-phospho-AKT (Cell Signal Tech, 4060 T, USA), anti-STAT3 (Abcam, ab68153, UK), anti-phospho-STAT3 (Abcam, ab76315, UK), anti-CD47 (Abcam, ab284132, UK), anti-ITGA5 (Abcam, ab150361, UK), anti-GAPDH (Proteintech, 60,004—1, USA). After washes with TBST, the membranes were incubated with HRP conjugated goat-anti-mouse (Beyotime, A0216, China) or goat-anti-rabbit (Beyotime, A0208, China) secondary antibodies. The immunoreactive bands were visualized with BeyoECL Plus (Beyotime, P0018S, China).

### Mouse castration-resistant prostate cancer model

Murine prostate cancer TRAMP-C1 cells (1 × 10^6^) were injected subcutaneously into C57BL/6 mice (male, 6-week-old, Beijing Huafukang Biotechnology Co., Ltd.). The tumor-bearing mice were castrated and randomly assigned to five groups. Animals were treated with C19-9N (5 mg/kg and 10 mg/kg), enzalutamide (10 mg/kg), enzalutamide (10 mg/kg) combined with C19-9N (10 mg/kg) or vehicle by orally administration once daily. The tumor volume and mouse body weight were measured every 2 days. Tumor weight obtained following termination of the experiment. Tumor volume measure was same as BALB/c-nude mice tumor growth xenograft model.

### In vivo subcutaneous tumor growth xenograft model

BALB/c-nude mice (male, 6-week-old) were obtained from Beijing Huafukang Biotechnology Co., Ltd. (Beijing, China) and housed in the animal care facility. The animal use protocol was approved by the Institutional Animal Care and Use Committee of Peking University Health Science Center. The 22RV1 xenograft tumor model was developed by subcutaneously injecting 1 × 10^6^ cells in suspension. When tumor nodules were developed to a volume of about 75 mm^3^, tumor-bearing mice were randomly assigned to four groups and treated with C19-9N (5 mg/kg and 10 mg/kg), enzalutamide (10 mg/kg) or vehicle by orally administration twice daily. The tumor volume and mouse body weight were measured twice a week. Tumor volume was computed by the following formula: Volume = (*d1* × *d2* × *d3*) × 0.5236, and *dn* means the three orthogonal diameter measurements.

### Human bone metastatic prostate cancer model

Intratibial injection PC3 PCa model was used to mimic the scenario when cancer cells transplanted to the bone microenvironment. After 7-day post tumor inoculation, BALB/C nude mice bearing luciferase-expressing PC3 tumors on the right tibiae were randomly divided into three groups. PBS, 25 mg/kg Saracatinib, or 10 mg/kg C19-9N or vehicle were administered orally twice daily. Tumor growth was monitored by detecting the luminescence intensity at the tumor sites. On day 32, the mice were sacrificed, and then the tumors were collected were fixed in 4% paraformaldehyde, embedded in paraffin and cut into 5 μm tissue sections.

### Mouse bone metastasis prostate cancer model

The bone metastasis model was established by intracardiac injection of MycCaP-Bo cells (4 × 10^5^ cells per mouse) into FVB (4-week-old, GemPharmatech, China) mice seven days prior to the start of the treatment (designated as Day −7). On Day 0, in vivo bioluminescence imaging (BLI) was performed to confirm the development of bone metastases. Mice with confirmed metastases were then randomized into different treatment groups based on their baseline BLI signal intensity, as outlined in the respective figures. PBS, 10 mg/kg enzalutamide, or 10 mg/kg C19-9N were administered orally twice daily. Metastatic growth was monitored via BLI twice weekly. Quantification of metastatic burden was primarily focused on the hindlimb regions. The BLI signal from each metastasis was normalized to its corresponding signal on Day 0 to calculate the relative tumor growth. Bioluminescence imaging was performed using an IVIS Lumina Series III (Perkinelmer, USA), and image radiance values were normalized using Living Image Software (Perkinelmer, USA).

### Patient-derived xenograft (PDX) model

PDX models were established using 4- to 6-week-old male NOG mice (Vital River Laboratories, China). A freshly resected neuroendocrine prostate cancer specimen (F0) was subcutaneously implanted into the dorsal flank of the mice to generate the first passage tumors (F1). Once the F1 tumor reached approximately 1000 mm^3^, the host mouse was euthanized, and the tumors were excised for subsequent passaging (F2 tumors). During this process, necrotic tissues were first removed, and the remaining viable tumor tissue was dissected into 2–3 mm^3^ tissue blocks. These blocks were then implanted into the dorsal flanks of new recipient NOG mice. Tumors from the F3 generation and beyond were preserved long-term in liquid nitrogen using CryoStor®CS10 cryopreservation medium (100—1061, StemCell Technologies). Tumor-bearing mice were randomly assigned to four groups and given docetaxel (10 mg/kg, once a week), cisplatin (5 mg/kg, once a week), C19-9N (20 mg/kg, twice a day) or vehicle for 3 weeks. The tumor volume and mouse body weight were measured twice a week. Thereafter, the mice were sacrificed and the tumor samples were dissected and isolated immediately. Docetaxel (HY-B0011) and cisplatin (HY-17394) were purchased from MCE (MedChemexpress, Shanghai, China).

### Toxicity studies in mice

A total of sixty 8-week-old BALB/c mice (30 males and 30 females, 25 ± 2 g) were randomly assigned to six groups with equal sex distribution. For the acute toxicity study, mice received a single oral gavage of C19-9N at doses of 50 mg/kg, 100 mg/kg or vehicle. In the long-term toxicity study, mice were orally dosed with C19-9N at 30 mg/kg, 60 mg/kg, or vehicle control, with treatments administered twice daily for a 14-day duration. General health conditions, body weight changes, and mortality were monitored and recorded throughout the experimental period. At the end of the experiment, all surviving mice were euthanized and autopsied.

### Single-cell RNA-seq data processing

Raw sequencing data generated from the 10 × Genomics platform were processed using Cell Ranger (v7.1.0) and aligned to the GRCm39 reference genome to obtain the UMI count matrix. The resulting matrix was imported into the R environment (v4.3.2) and processed using Seurat (v5.3.0). Doublets were identified and removed using scDblFinder (v1.16.0). Cells expressing fewer than 200 or more than 7000 genes, or with > 15% mitochondrial transcript content, were excluded from downstream analyses. After filtering low-quality cells, the data were normalized and scaled. Batch effects among samples were corrected using the mutual nearest neighbors (MNN) algorithm implemented in Seurat, yielding an integrated expression matrix for subsequent analyses and visualization. Highly variable genes (HVGs) were identified, and the top 2000 HVGs were selected for principal component analysis (PCA). The first 30 principal components (PCs) were used for clustering analysis and dimensionality reduction.

### Cell type annotation and Gene Ontology (GO) enrichment analysis

Cell type annotation was performed based on the expression patterns of canonical marker genes. Differentially expressed genes for each subset were identified using the FindAllMarkers function in Seurat with default parameters. Genes were considered significant if they met all the following criteria: adjusted p value < 0.05, average log fold change (logFC) > 0.25, and expression in > 25% of cells within the cluster. Following cell subset annotation, subset-specific marker genes were subjected to Gene Ontology (GO) enrichment analysis using clusterProfiler (v4.10.1). A q-value threshold of 0.05 was applied to identify significantly enriched functional pathways. Enrichment results were visualized to highlight key biological processes associated with each cell subset.

### Statistics

All quantitative data are presented as the mean ± standard deviation (SD) from at least three independent experiments unless otherwise indicated. Differences between two groups were assessed using two-tailed Student’s t-tests. Survival curves were generated using the Kaplan–Meier method and compared using the log-rank test, and hazard ratios were estimated by univariate and multivariate Cox proportional hazards regression analyses. P values < 0.05 were considered statistically significant.

## Supplementary Information

Supplementary Material 1.

Supplementary Material 2.

## Data Availability

The datasets used and analysed during the current study are available from the corresponding author on reasonable request.

## References

[1] Vaccarella S, Li M, Bray F, Kvale R, Serraino D, Lorenzoni V, et al. Prostate cancer incidence and mortality in Europe and implications for screening activities: population based study. BMJ. 2024;386:e077738. 10.1136/bmj-2023-077738. 39231588 10.1136/bmj-2023-077738 PMC11372856

[2] Kratzer TB, Mazzitelli N, Star J, Dahut WL, Jemal A, Siegel RL. Prostate cancer statistics, 2025. CA Cancer J Clin. 2025;75:485–97. 10.3322/caac.70028. 40892160 10.3322/caac.70028 PMC12593258

[3] Cheng Q, Butler W, Zhou Y, Zhang H, Tang L, Perkinson K, et al. Pre-existing castration-resistant prostate cancer-like cells in primary prostate cancer promote resistance to hormonal therapy. Eur Urol. 2022;81:446–55. 10.1016/j.eururo.2021.12.039. 35058087 10.1016/j.eururo.2021.12.039 PMC9018600

[4] Feng DC, Zhu WZ, Wang J, Li DX, Shi X, Xiong Q, You J, Han P, Qiu S, Wei Q, Yang L. The implications of single-cell RNA-seq analysis in prostate cancer: unraveling tumor heterogeneity, therapeutic implications and pathways towards personalized therapy. Military Med Res. 2024;11. 10.1186/s40779-024-00526-7. 10.1186/s40779-024-00526-7 PMC11007901 38605399

[5] Song H, Weinstein HNW, Allegakoen P, Wadsworth MH, Xie J, Yang H, et al. Single-cell analysis of human primary prostate cancer reveals the heterogeneity of tumor-associated epithelial cell states. Nat Commun. 2022. 10.1038/s41467-021-27322-4. 35013146 10.1038/s41467-021-27322-4 PMC8748675

[6] Lundberg A, Zhang M, Aggarwal R, Li H, Zhang L, Foye A, et al. The genomic and epigenomic landscape of double-negative metastatic prostate cancer. Cancer Res. 2023;83:2763–74. 10.1158/0008-5472.can-23-0593. 37289025 10.1158/0008-5472.CAN-23-0593 PMC10425725

[7] Wang Y, Wang Y, Ci X, Choi SYC, Crea F, Lin D, et al. Molecular events in neuroendocrine prostate cancer development. Nat Rev Urol. 2021;18:581–96. 10.1038/s41585-021-00490-0. 34290447 10.1038/s41585-021-00490-0 PMC10802813

[8] Beltran H, Rickman DS, Park K, Chae SS, Sboner A, MacDonald TY, et al. Molecular characterization of neuroendocrine prostate cancer and identification of new drug targets. Cancer Discov. 2011;1:487–95. 10.1158/2159-8290.cd-11-0130. 22389870 10.1158/2159-8290.CD-11-0130 PMC3290518

[9] Gasparri AM, Pocaterra A, Colombo B, Taiè G, Gnasso C, Gori A, et al. Blockade of αvβ6 and αvβ8 integrins with a chromogranin A-derived peptide inhibits TGFβ activation in tumors and suppresses tumor growth. J Exp Clin Cancer Res. 2025. 10.1186/s13046-025-03352-4. 40055773 10.1186/s13046-025-03352-4 PMC11889887

[10] Drivalos A, Emmanouil G, Gavriatopoulou M, Terpos E, Sergentanis TN, Psaltopoulou T. Integrin expression in correlation to clinicopathological features and prognosis of prostate cancer: a systematic review and meta-analysis. Urol Oncol. 2021;39:221–32. 10.1016/j.urolonc.2020.12.024. 33558138 10.1016/j.urolonc.2020.12.024

[11] Pang X, He X, Qiu Z, Zhang H, Xie R, Liu Z, Gu Y, Zhao N, Xiang Q, Cui Y. Targeting integrin pathways: mechanisms and advances in therapy. Signal Transduct Target Ther. 2023;8. 10.1038/s41392-022-01259-6. 10.1038/s41392-022-01259-6 PMC9805914 36588107

[12] Sutherland M, Gordon A, Shnyder S, Patterson L, Sheldrake H. RGD-binding integrins in prostate cancer: expression patterns and therapeutic prospects against bone metastasis. Cancers. 2012;4:1106–45. 10.3390/cancers4041106. 24213501 10.3390/cancers4041106 PMC3712721

[13] Lu H, Wang T, Li J, Fedele C, Liu Q, Zhang J, et al. αvβ6 integrin promotes castrate-resistant prostate cancer through JNK1-mediated activation of androgen receptor. Cancer Res. 2016;76:5163–74. 10.1158/0008-5472.can-16-0543. 27450452 10.1158/0008-5472.CAN-16-0543 PMC5012867

[14] Zheng DQ, Woodard AS, Fornaro M, Tallini G, Languino LR. Prostatic carcinoma cell migration via alpha(v)beta3 integrin is modulated by a focal adhesion kinase pathway. Cancer Res. 1999;59:1655–64. 10197643

[15] Damiano J. Integrins as novel drug targets for overcoming innate drug resistance. Curr Cancer Drug Targets. 2002;2:37–43. 10.2174/1568009023334033. 12188919 10.2174/1568009023334033

[16] Quaglia F, Krishn SR, Daaboul GG, Sarker S, Pippa R, Domingo-Domenech J, Kumar G, Fortina P, McCue P, Kelly WK, et al. Small extracellular vesicles modulated by αVβ3 integrin induce neuroendocrine differentiation in recipient cancer cells. J Extracell Vesicles. 2020;9. 10.1080/20013078.2020.1761072. 10.1080/20013078.2020.1761072 PMC7448905 32922691

[17] Krishn SR, Singh A, Bowler N, Duffy AN, Friedman A, Fedele C, et al. Prostate cancer sheds the αvβ3 integrin in vivo through exosomes. Matrix Biol. 2019;77:41–57. 10.1016/j.matbio.2018.08.004. 30098419 10.1016/j.matbio.2018.08.004 PMC6541230

[18] Hamidi H, Ivaska J. Every step of the way: integrins in cancer progression and metastasis. Nat Rev Cancer. 2018;18:533–48. 10.1038/s41568-018-0038-z. 30002479 10.1038/s41568-018-0038-z PMC6629548

[19] McCabe NP, De S, Vasanji A, Brainard J, Byzova TV. Prostate cancer specific integrin αvβ3 modulates bone metastatic growth and tissue remodeling. Oncogene. 2007;26:6238–43. 10.1038/sj.onc.1210429. 17369840 10.1038/sj.onc.1210429 PMC2753215

[20] Brown NF, Marshall JF. Integrin-mediated TGFβ activation modulates the tumour microenvironment. Cancers. 2019;11:1221. 10.3390/cancers11091221. 31438626 10.3390/cancers11091221 PMC6769837

[21] Verrillo CE, Quaglia F, Shields CD, Lin S, Kossenkov AV, Tang HY, et al. Expression of the αVβ3 integrin affects prostate cancer sEV cargo and density and promotes sEV pro‐tumorigenic activity in vivo through a GPI‐anchored receptor, NgR2. J Extracell Vesicles. 2024. 10.1002/jev2.12482. 39105261 10.1002/jev2.12482 PMC11301027

[22] Lu H, Bowler N, Harshyne LA, Craig Hooper D, Krishn SR, Kurtoglu S, et al. Exosomal αvβ6 integrin is required for monocyte M2 polarization in prostate cancer. Matrix Biol. 2018;70:20–35. 10.1016/j.matbio.2018.03.009. 29530483 10.1016/j.matbio.2018.03.009 PMC6081240

[23] Trerotola M, Jernigan DL, Liu Q, Siddiqui J, Fatatis A, Languino LR. Trop-2 promotes prostate cancer metastasis by modulating β1 Integrin functions. Cancer Res. 2013;73:3155–67. 10.1158/0008-5472.can-12-3266. 23536555 10.1158/0008-5472.CAN-12-3266 PMC3655712

[24] Thomas R, Jerome JM, Dang TD, Souto EP, Mallam JN, Rowley DR. Androgen receptor variant-7 regulation by tenascin-c induced src activation. Cell Commun Signal. 2022;20. 10.1186/s12964-022-00925-0. 10.1186/s12964-022-00925-0 PMC9364530 35948987

[25] Li X-F, Selli C, Zhou H-L, Cao J, Wu S, Ma R-Y, et al. Macrophages promote anti-androgen resistance in prostate cancer bone disease. J Exp Med. 2023. 10.1084/jem.20221007. 36749798 10.1084/jem.20221007 PMC9948761

[26] Pang X, Sun X, Gu Y, He X, Gong K, Song S, Zhang J, Xia J, Liu Z, Cui Y. Discovery of C19–9 as a novel non-RGD inhibitor of αvβ3 to overcome enzalutamide resistance in castration-resistant prostate cancer. Signal Transduct Target Ther. 2023;8. 10.1038/s41392-022-01236-z. 10.1038/s41392-022-01236-z PMC9911763 36759595

[27] Zhang M, Latham DE, Delaney MA, Chakravarti A. Survivin mediates resistance to antiandrogen therapy in prostate cancer. Oncogene. 2005;24:2474–82. 10.1038/sj.onc.1208490. 15735703 10.1038/sj.onc.1208490

[28] Wang T, Huang J, Vue M, Alavian MR, Goel HL, Altieri DC, et al. αvβ3 Integrin mediates radioresistance of prostate cancer cells through regulation of Survivin. Mol Cancer Res. 2019;17:398–408. 10.1158/1541-7786.mcr-18-0544. 30266752 10.1158/1541-7786.MCR-18-0544 PMC6359981

[29] Li C, Qiu S, Liu X, Guo F, Zhai J, Li Z, Deng L, Ge L, Qian H, Yang L, Xu B. Extracellular matrix-derived mechanical force governs breast cancer cell stemness and quiescence transition through integrin-DDR signaling. Signal Transduct Target Ther. 2023;8. 10.1038/s41392-023-01453-0. 10.1038/s41392-023-01453-0 PMC10300038 37369642

[30] Lang SH, Sharrard RM, Stark M, Villette JM, Maitland NJ. Prostate epithelial cell lines form spheroids with evidence of glandular differentiation in three-dimensional Matrigel cultures. Br J Cancer. 2001;85:590–9. 10.1054/bjoc.2001.1967. 11506501 10.1054/bjoc.2001.1967 PMC2364090

[31] Tatar C, Avci CB, Acikgoz E, Oktem G. Doxorubicin-induced senescence promotes resistance to cell death by modulating genes associated with apoptotic and necrotic pathways in prostate cancer DU145 CD133+/CD44+ cells. Biochem Biophys Res Commun. 2023;680:194–210. 10.1016/j.bbrc.2023.09.032. 37748252 10.1016/j.bbrc.2023.09.032

[32] Xie C, Wang Z, Ba Y, Aguilar J, Kyan A, Zhong L, et al. BMP signaling inhibition overcomes chemoresistance of prostate cancer. Am J Cancer Res. 2023;13:4073–86. 37818054 PMC10560954

[33] Huang Y, Hong W, Wei X. The molecular mechanisms and therapeutic strategies of EMT in tumor progression and metastasis. J Hematol Oncol. 2022;15. 10.1186/s13045-022-01347-8. 10.1186/s13045-022-01347-8 PMC9461252 36076302

[34] Antonarakis ES, Lu C, Wang H, Luber B, Nakazawa M, Roeser JC, et al. AR-V7 and resistance to Enzalutamide and Abiraterone in prostate cancer. N Engl J Med. 2014;371:1028–38. 10.1056/nejmoa1315815. 25184630 10.1056/NEJMoa1315815 PMC4201502

[35] Kuzuoglu-Ozturk D, Nguyen HG, Xue L, Figueredo E, Subramanyam V, Liu I, et al. Small-molecule RNA therapeutics to target prostate cancer. Cancer Cell. 2025;43:841-855.e848. 10.1016/j.ccell.2025.02.027. 40118049 10.1016/j.ccell.2025.02.027 PMC12124822

[36] Ma L, Tian Y, Qian T, Li W, Liu C, Chu B, Kong Q, Cai R, Bai P, Ma L, et al. Kindlin-2 promotes Src-mediated tyrosine phosphorylation of androgen receptor and contributes to breast cancer progression. Cell Death Dis. 2022;13. 10.1038/s41419-022-04945-z. 10.1038/s41419-022-04945-z PMC9122951 35595729

[37] Yu J, Gao Y, Zhang M, Gao Y, Wang C, Niu Y, et al. FMNL2/SRC-mediated androgen receptor translocation into the nucleus promotes enzalutamide resistance of prostate cancer. iScience. 2025;28:112205. 10.1016/j.isci.2025.112205. 40212590 10.1016/j.isci.2025.112205 PMC11985155

[38] Li J, Qiu H, Dong Q, Yu H, Piao C, Li Z, Sun Y, Cui X. Androgen-targeted hsa_circ_0085121 encodes a novel protein and improves the development of prostate cancer through facilitating the activity of PI3K/Akt/mTOR pathway and enhancing AR-V7 alternative splicing. Cell Death Dis. (2024;15. 10.1038/s41419-024-07246-9. 10.1038/s41419-024-07246-9 PMC11579034 39567496

[39] Liu C, Lou W, Armstrong C, Zhu Y, Evans CP, Gao AC. Niclosamide suppresses cell migration and invasion in enzalutamide resistant prostate cancer cells via Stat3-AR axis inhibition. Prostate. 2015;75:1341–53. 10.1002/pros.23015. 25970160 10.1002/pros.23015 PMC4536195

[40] Han M, Li F, Zhang Y, Dai P, He J, Li Y, et al. FOXA2 drives lineage plasticity and KIT pathway activation in neuroendocrine prostate cancer. Cancer Cell. 2022;40:1306-1323.e1308. 10.1016/j.ccell.2022.10.011. 36332622 10.1016/j.ccell.2022.10.011

[41] Sun Y, Ren S, Wen W, Jing J, Luo X, Shao S, Duan R, Zeng G, Guo J. Targeting CXCR2 in prostate cancer cells can block CD47-SIRPα interaction and reverse M2 macrophage polarization in the TME. Mol Cancer. 2025;24. 10.1186/s12943-025-02436-1. 10.1186/s12943-025-02436-1 PMC12574227 41163221

[42] Cho S, Rhee S, Madl CM, Caudal A, Thomas D, Kim H, et al. Selective inhibition of stromal mechanosensing suppresses cardiac fibrosis. Nature. 2025;642:766–75. 10.1038/s41586-025-08945-9. 40307543 10.1038/s41586-025-08945-9 PMC12176515

[43] Oliveira MFd, Romero JP, Chung M, Williams SR, Gottscho AD, Gupta A, et al. High-definition spatial transcriptomic profiling of immune cell populations in colorectal cancer. Nat Genet. 2025;57:1512–23. 10.1038/s41588-025-02193-3. 40473992 10.1038/s41588-025-02193-3 PMC12165841

[44] Yu G, Corn PG, Mak CSL, Liang X, Zhang M, Troncoso P, et al. Prostate cancer-induced endothelial-cell-to-osteoblast transition drives immunosuppression in the bone-tumor microenvironment through Wnt pathway-induced M2 macrophage polarization. Proc Natl Acad Sci U S A. 2024;121:e2402903121. 10.1073/pnas.2402903121. 39102549 10.1073/pnas.2402903121 PMC11331113

[45] Morrissey MA, Kern N, Vale RD. CD47 ligation repositions the inhibitory receptor SIRPA to suppress Integrin activation and phagocytosis. Immunity. 2023;56:2172. 10.1016/j.immuni.2023.08.003. 37703830 10.1016/j.immuni.2023.08.003 PMC10593492

[46] Girnius N, Henstridge AZ, Marks B, Yu JK, Gray GK, Sander C, et al. Cilengitide sensitivity is predicted by overall integrin expression in breast cancer. Breast Cancer Res. 2024;26:187. 10.1186/s13058-024-01942-2. 39707454 10.1186/s13058-024-01942-2 PMC11660856

[47] Stojanović N, Dekanić A, Paradžik M, Majhen D, Ferenčak K, Ruščić J, et al. Differential effects of Integrin αv knockdown and Cilengitide on sensitization of Triple-Negative Breast Cancer and Melanoma cells to microtubule poisons. Mol Pharmacol. 2018;94:1334–51. 10.1124/mol.118.113027. 30262596 10.1124/mol.118.113027

[48] Truong HH, Xiong J, Ghotra VP, Nirmala E, Haazen L, Le Dévédec SE, et al. β1 integrin inhibition elicits a prometastatic switch through the TGFβ-miR-200-ZEB network in E-cadherin-positive triple-negative breast cancer. Sci Signal. 2014;7:ra15. 10.1126/scisignal.2004751. 24518294 10.1126/scisignal.2004751

[49] Soupir AC, Hayes MT, Peak TC, Ospina O, Chakiryan NH, Berglund AE, et al. Increased spatial coupling of integrin and collagen IV in the immunoresistant clear-cell renal-cell carcinoma tumor microenvironment. Genome Biol. 2024. 10.1186/s13059-024-03435-z. 39639369 10.1186/s13059-024-03435-z PMC11622564

[50] Morrissey C, Vessella RL. The role of tumor microenvironment in prostate cancer bone metastasis. J Cell Biochem. 2007;101:873–86. 10.1002/jcb.21214. 17387734 10.1002/jcb.21214

[51] Yu PC, Yue CX, Dong WZ, Hao CY, Qiao YF, Liu D, et al. Cancer immunotherapy via disruption of Integrin αvβ3 and CD47 costabilization on cancer cell surface. Adv Sci. 2025. 10.1002/advs.202501602. 10.1002/advs.202501602 PMC12786276 41168993

