# Supplementary material for: An orally bioavailable pan-αv/α5β1 integrin antagonist prevents aggressive prostate cancer progression via suppressing both oncogenic signals and CD47-mediated immune escape
Source: Mol Cancer. 2026 May 13;25:166. doi: 10.1186/s12943-026-02686-7 (PMC13343748; doi:10.1186/s12943-026-02686-7)
Supplement: Supplementary file 1 — Supplementary Material 1. [file 12943_2026_2686_MOESM1_ESM.pdf]

Supplementary Materials

Figure S1

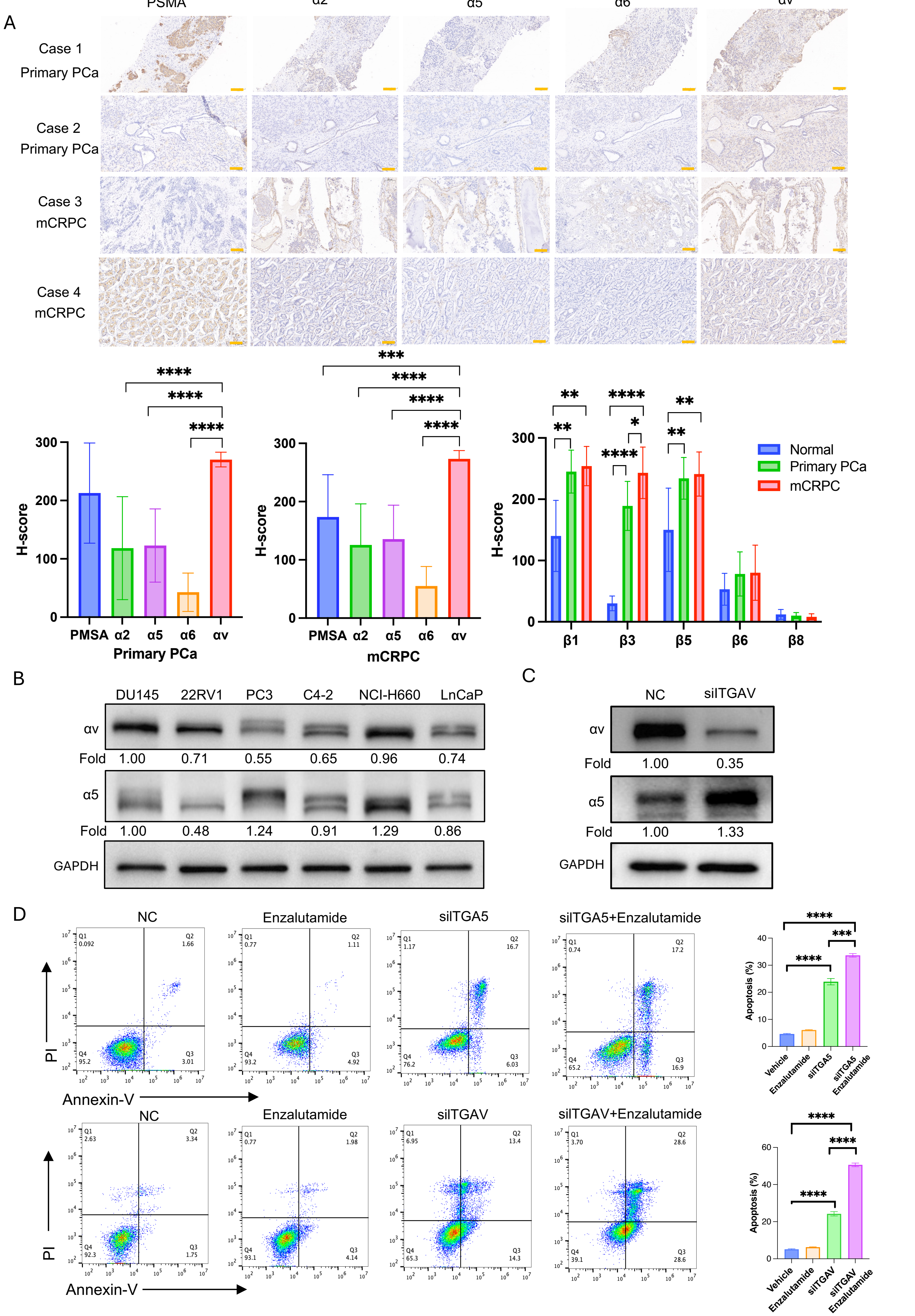
A
PSMA
α2
α5
α6
αv
Case 1
Primary PCa
Case 2
Primary PCa
Case 3
mCRPC
Case 4
mCRPC
H-score
PMSA α2 α5 α6 αv
Primary PCa
mCRPC
β1 β3 β5 β6 β8
Normal
Primary PCa
mCRPC
B
DU145 22RV1 PC3 C4-2 NCI-H660 LnCaP
αv
Fold 1.00 0.71 0.55 0.65 0.96 0.74
α5
Fold 1.00 0.48 1.24 0.91 1.29 0.86
GAPDH
C
NC siITGAV
αv
Fold 1.00 0.35
α5
Fold 1.00 1.33
GAPDH
D
NC
Enzalutamide
siITGA5
siITGA5+Enzalutamide
siITGAV
siITGAV+Enzalutamide
PI
Annexin-V
Apoptosis (%)
Vehicle
Enzalutamide
siITGA5
siITGA5 Enzalutamide
siITGAV
siITGAV Enzalutamide

Figure S1. Integrin αv/α5 are overexpressed in mCRPC and their silencing enhances enzalutamide-induced apoptosis.

(A) Integrin subunit expression in primary PCa and mCRPC tissues. Top: Representative IHC images of PSMA (PCa marker) and integrin subunits in primary PCa and mCRPC tissues (Scale bar = 100 μm). Bottom: Quantitative H-score analysis of integrin subunits in primary Pca, mCRPC and normal prostate tissues. The H-score is calculated as: (1 × % weakly stained cells) + (2 × % moderately stained cells) + (3 × % strongly stained cells) .

(B) Integrin αv/α5 expression in PCa cell lines. Western blot of integrin αv and α5 protein levels in PCa cell lines (DU145, 22RV1, PC3, C4-2, NCI-H660, LnCap).

(C) Compensatory regulation of integrin αv and α5 protein expression. Western blot analysis of ITGAV (αv) and ITGA5 (α5) protein expression in in 22RV1 cells transfected with siRNA targeting ITGAV (siITGAV).

(D) αv/α5 silencing enhances enzalutamide-induced apoptosis. Flow cytometry analysis and quantitative analysis of apoptosis rate (Annexin V-FITC^{+}) in 22RV1 cells treated with Enzalutamide ± siRNA targeting αv (siITGAV) or α5 (siITGA5). Data are expressed as mean ± SD, *P-value < 0.05, **P-value <0.01, ***P-value < 0.001.

Figure S2

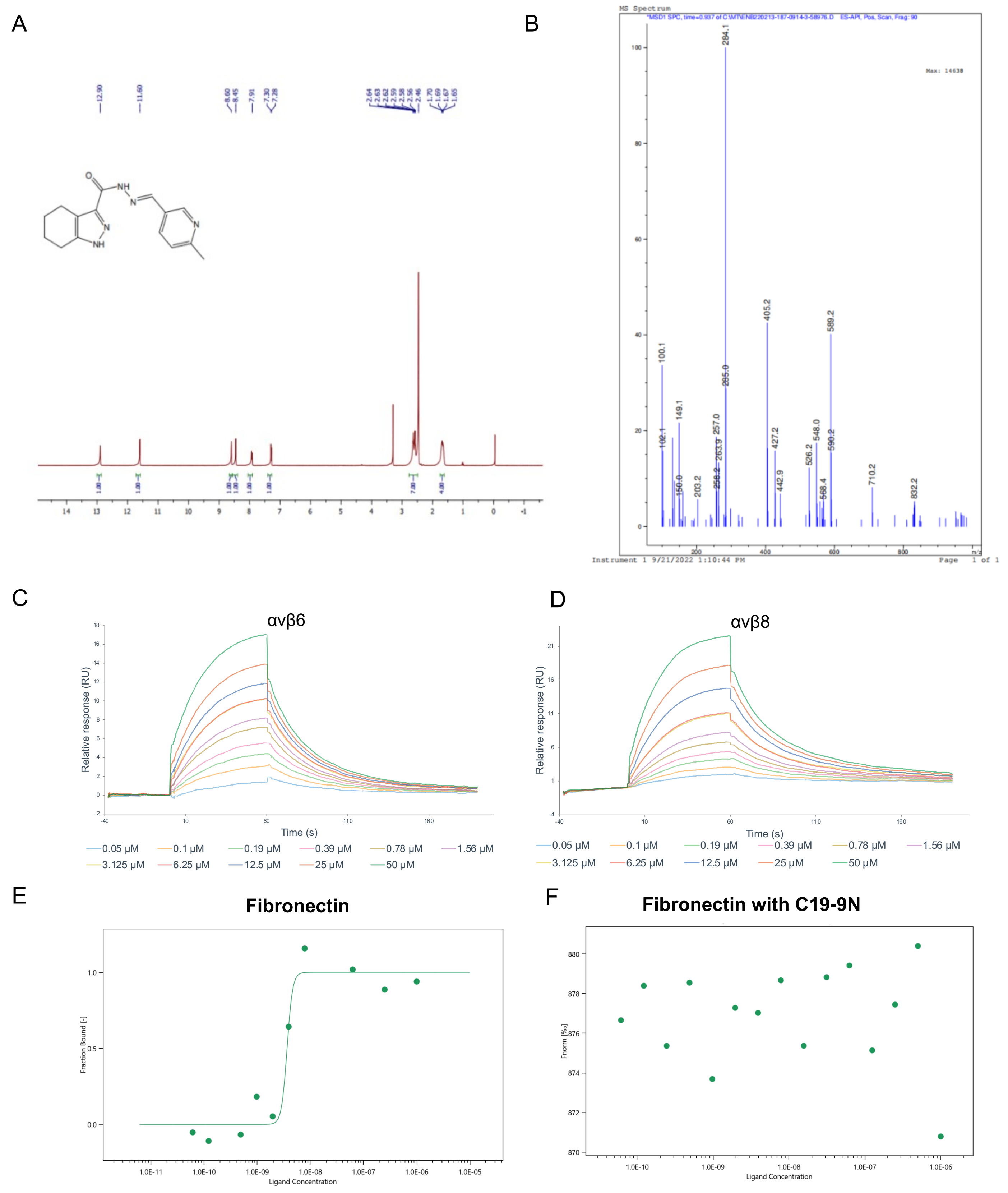
A
12.90
11.60
8.60
8.45
7.91
7.30
7.28
2.64
2.63
2.62
2.59
2.58
2.56
2.46
1.70
1.69
1.67
1.65
O
NH
N
N
NH
N
14 13 12 11 10 9 8 7 6 5 4 3 2 1 0 -1
B
MS Spectrum
Max: 14638
284.1
405.2
589.2
100.1
285.0
149.1
257.0
102.1
427.2
526.2
548.0
590.2
263.9
258.2
203.2
150.0
442.9
568.4
710.2
832.2
Instrument 1 9/21/2022 1:10:44 PM
Page 1 of 1
C
αvβ6
Relative response (RU)
Time (s)
0.05 μM
0.1 μM
0.19 μM
0.39 μM
0.78 μM
1.56 μM
3.125 μM
6.25 μM
12.5 μM
25 μM
50 μM
D
αvβ8
Relative response (RU)
Time (s)
0.05 μM
0.1 μM
0.19 μM
0.39 μM
0.78 μM
1.56 μM
3.125 μM
6.25 μM
12.5 μM
25 μM
50 μM
E
Fibronectin
Fraction Bound [-]
Ligand Concentration
F
Fibronectin with C19-9N
Fnorm [‰]
Ligand Concentration

Figure S2. Structural characterization and integrin-binding affinity of C19-9N.

(A, B) Chemical structure and HPLC-MS characterization of C19-9N.

(C, D) C19-9N binds to integrin αvβ6 and αvβ8 with dose-dependent affinity measured via SPR.

(E) MST assay shows the binding of fibronectin to immobilized integrin αvβ3 in the absence of C19-9N.

(F) C19-9N inhibits fibronectin-integrin binding. MST test shows the abrogated binding of fibronectin to integrin in the presence of 5 μM C19-9N, confirming C19-9N blocks integrin-ligand interaction.

Figure S3

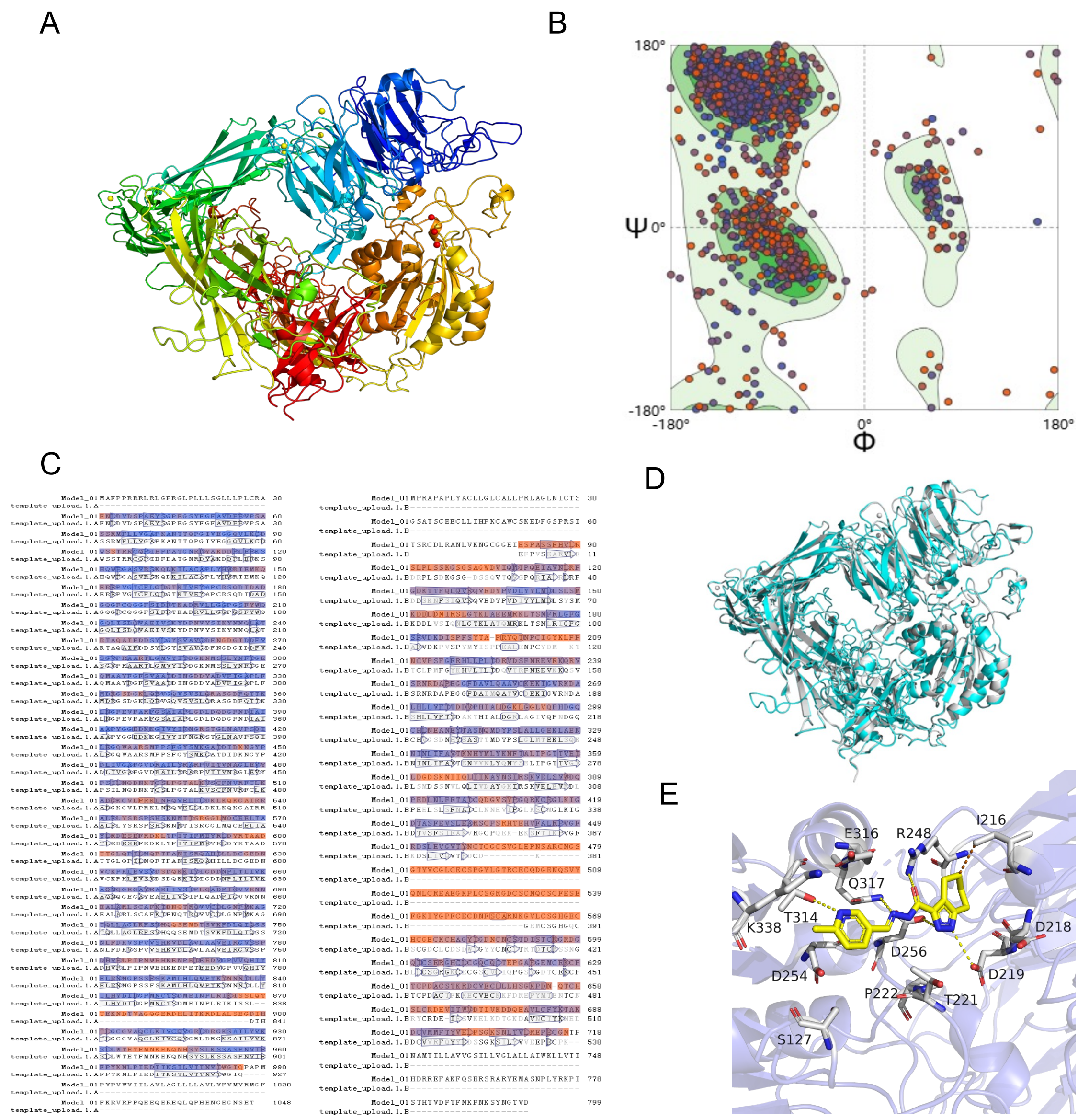

Figure S3. The homology mode and structural analysis of human integrin αvβ5 modeling results.

(A) The homology model of human integrin αvβ5.

(B) Ramachandran plot for human integrin αvβ5. Green regions represent residues in allowed regions, and white regions represent the residues in irrational regions.

(C) The structure-based sequence comparison between human integrin αv and the template structure. The structure-based sequence comparison between human integrin β5 and the template structure. The same or similar residues were highlighted in blue and dissimilar ones were highlighted in orange, the darker color indicated more similar or dissimilar residues.

(D) Homology model of human αvβ5.

(E) αvβ6-C19-9N complex, four hydrogen bonds were formed with T314, Q317, R248, and D219, alongside hydrophobic interactions with I216.

Figure S4

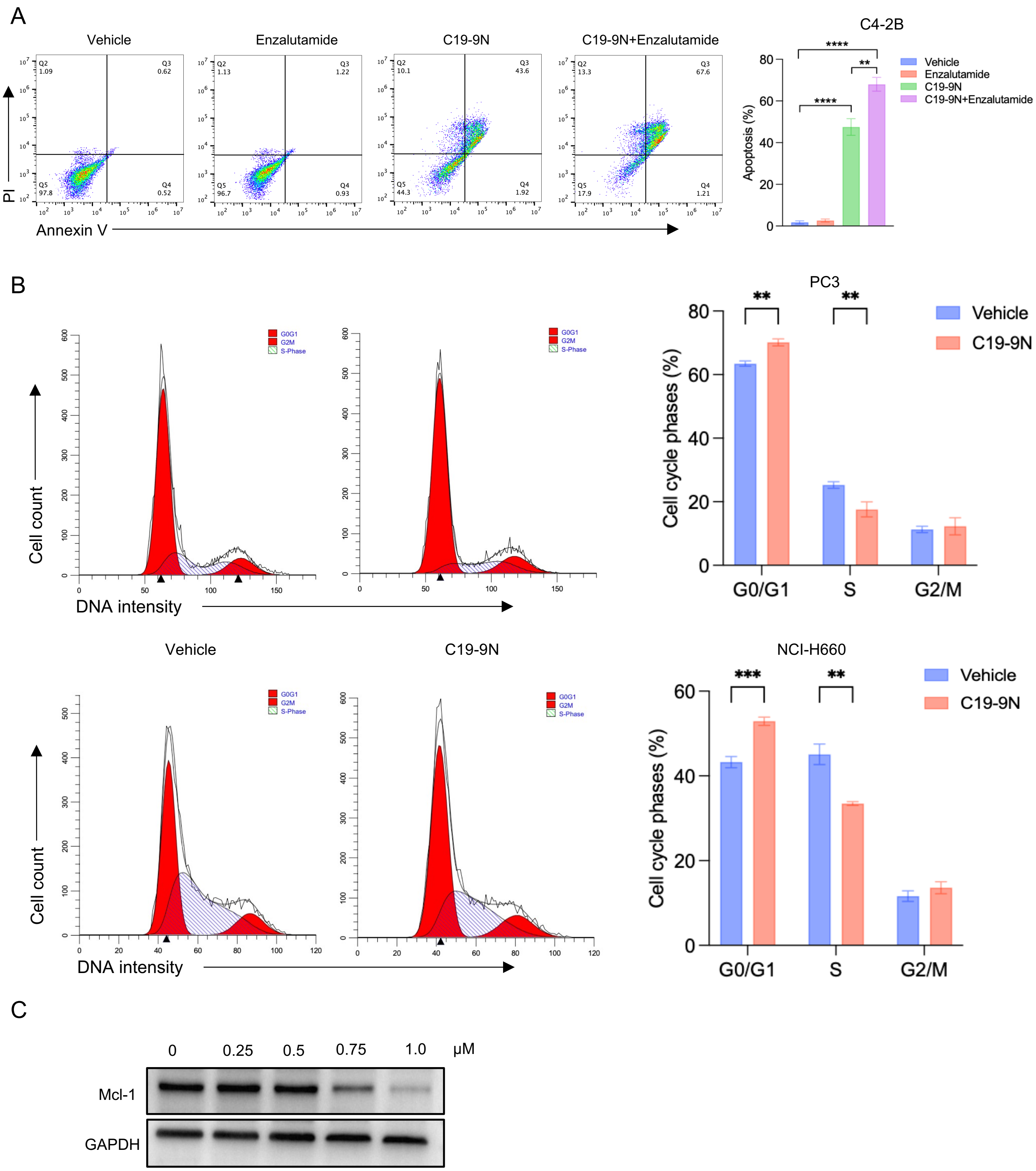

A
Vehicle
Enzalutamide
C19-9N
C19-9N+Enzalutamide
Q2 1.09
Q3 0.62
Q5 97.8
Q4 0.52
Q2 1.13
Q3 1.22
Q5 96.7
Q4 0.93
Q2 10.1
Q3 43.6
Q5 44.3
Q4 1.92
Q2 13.3
Q3 67.6
Q5 17.9
Q4 1.21
PI
Annexin V
C4-2B
Apoptosis (%)
****
**
****
Vehicle
Enzalutamide
C19-9N
C19-9N+Enzalutamide
B
G0G1
G2M
S-Phase
Cell count
DNA intensity
PC3
Cell cycle phases (%)
Vehicle
C19-9N
G0/G1
S
G2/M
**
**
Vehicle
C19-9N
NCI-H660
***
**
C
0
0.25
0.5
0.75
1.0
μM
Mcl-1
GAPDH

Figure S4. C19-9N enhances enzalutamide-induced apoptosis and alters cell cycle progression.

(A) C19-9N enhances enzalutamide-induced apoptosis in C4-2B cells. Representative flow cytometry dot plots showing apoptotic cells (Annexin V-FITC$^+$/PI$^-$ or Annexin V-FITC$^+$/PI$^+$) in cells treated with vehicle, enzalutamide, C19-9N, or C19-9N combined with enzalutamide. Right panel: Quantitative analysis of apoptosis rate.

(B) The effect of C19-19N on cell cycle analyzed by DNA content staining in PC3 and NCI-H660 cells . Left panels: Representative flow cytometry histograms (DNA intensity) showing cell cycle distribution (G0/G1, S, G2/M phases) in vehicle or C19-9N treated cells. Right panel: Quantitative analysis of cell cycle phase percentages.

(C) Representative blots showing the expression of Mcl-1 in cells treated with increasing concentrations of C19-9N (0–1 μM). Data are expressed as mean ± SD, *P-value < 0.05, **P-value <0.01, ***P-value < 0.001.

Figure S5

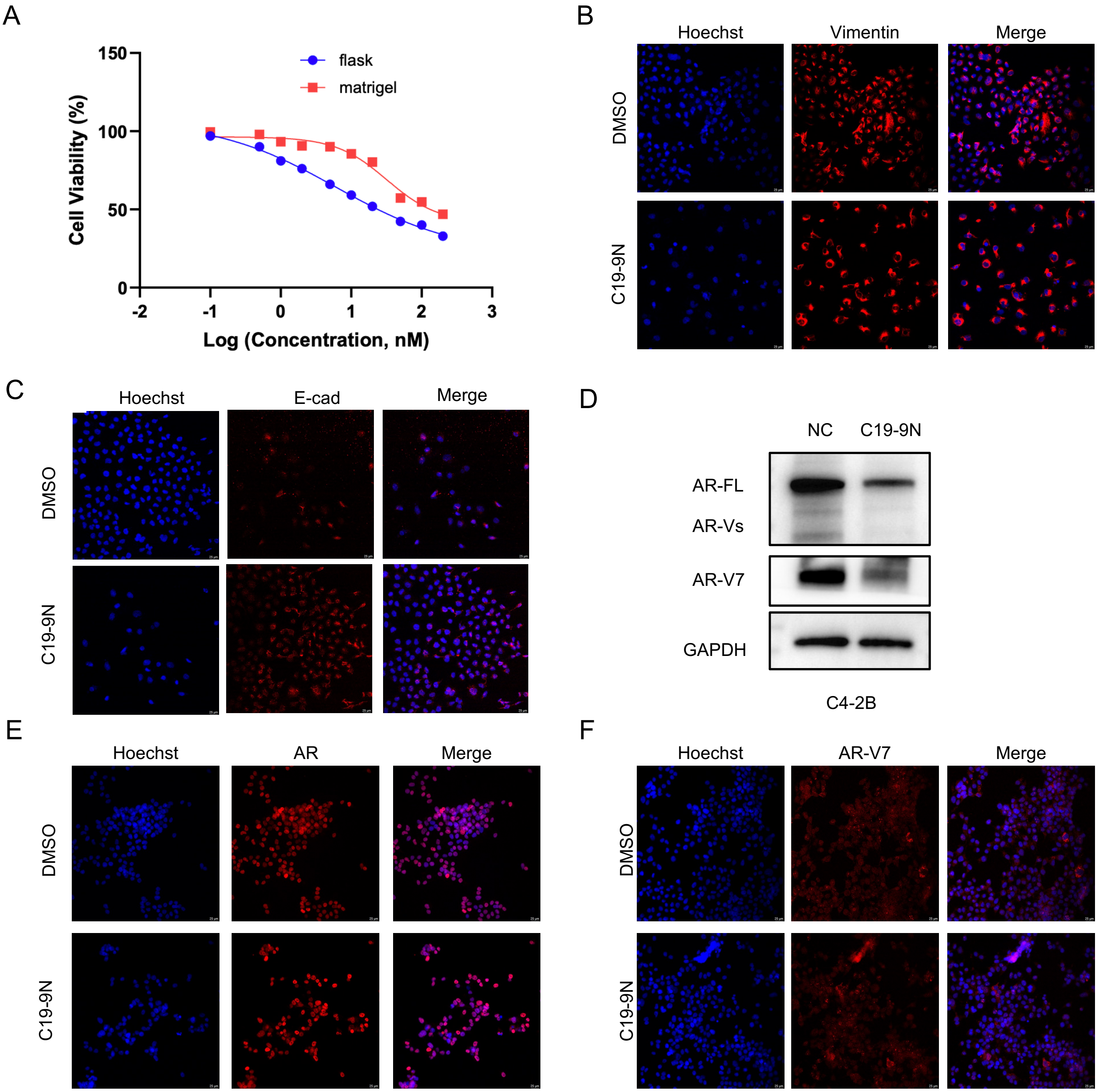
A
Cell Viability (%)
150
100
50
0
flask
matrigel
-2
-1
0
1
2
3
Log (Concentration, nM)
B
Hoechst
Vimentin
Merge
DMSO
C19-9N
C
Hoechst
E-cad
Merge
DMSO
C19-9N
D
NC
C19-9N
AR-FL
AR-Vs
AR-V7
GAPDH
C4-2B
E
Hoechst
AR
Merge
DMSO
C19-9N
F
Hoechst
AR-V7
Merge
DMSO
C19-9N

Figure S5. C19-9N reverses epithelial-mesenchymal transition (EMT), and downregulates AR/AR-V7 signaling in prostate cancer cells..

(A) Dose-response curves of cell viability in DU145 cultured in standard 2D formats (flask) versus 3D environments (matrigel) following treatment with varying concentrations of C19-9N.

(B, C) C19-9N reverses the epithelial-mesenchymal transition (EMT) phenotype in DU145-DR cells. C19-9N decreases mesenchymal marker Vimentin. Immunofluorescence staining (Hoechst: nuclear, Vimentin: mesenchymal marker) of DU145-DR cells. C19-9N increases epithelial marker E-cadherin. Immunofluorescence staining (Hoechst: nuclear, E-cad: E-cadherin) of DU145-DR cells. Scale bar = 10 μm.

(D) Downregulation of AR isoforms. Western blot analysis of C4-2B cell lysates following C19-9N treatment. C19-9N treatment markedly downregulated the protein levels of both Androgen Receptor full-length (AR-FL) and the splice variant AR-V7.

(E, F) Reduction of nuclear AR and AR-V7. Immunofluorescence staining of C4-2B cells treated with Vehicle or C19-9N. Representative images show that C19-9N treatment significantly reduced the nuclear accumulation of both AR and AR-V7 (red) compared to the control group. Nuclei were counterstained with Hoechst (blue). Data are expressed as mean ± SD, *P-value < 0.05, **P-value <0.01, ***P-value < 0.001.

Figure S6

A

	1 mg/kg (IV)	10 mg/kg (PO)
$T_{1/2}$ (min)	16.60±8.58	68.765±0.95
T_{max} (min)		15
C_{max} (ng/ml)	1296.0±176.67	561.7±70.0
$AUC_{0\text{-}t}$ (ng·min/mL)	17362.4±2507.1	81822.7±8872.5
$AUC_{0\text{-}\infty}$ (ng·min/mL)	18894.7±2547.4	81844.7±8884.4
CL (L/min/kg)	0.054±0.007	
F (%)		43.3

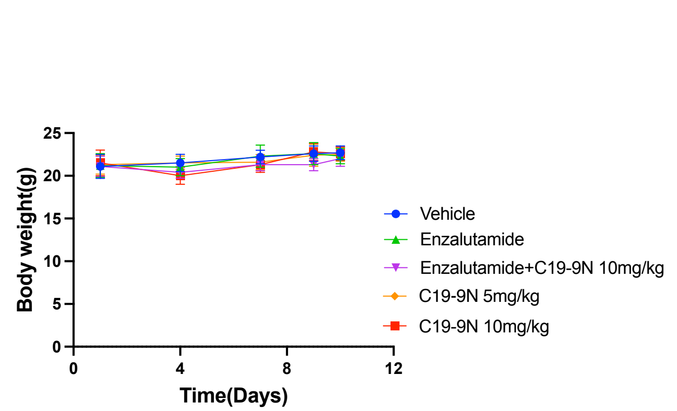

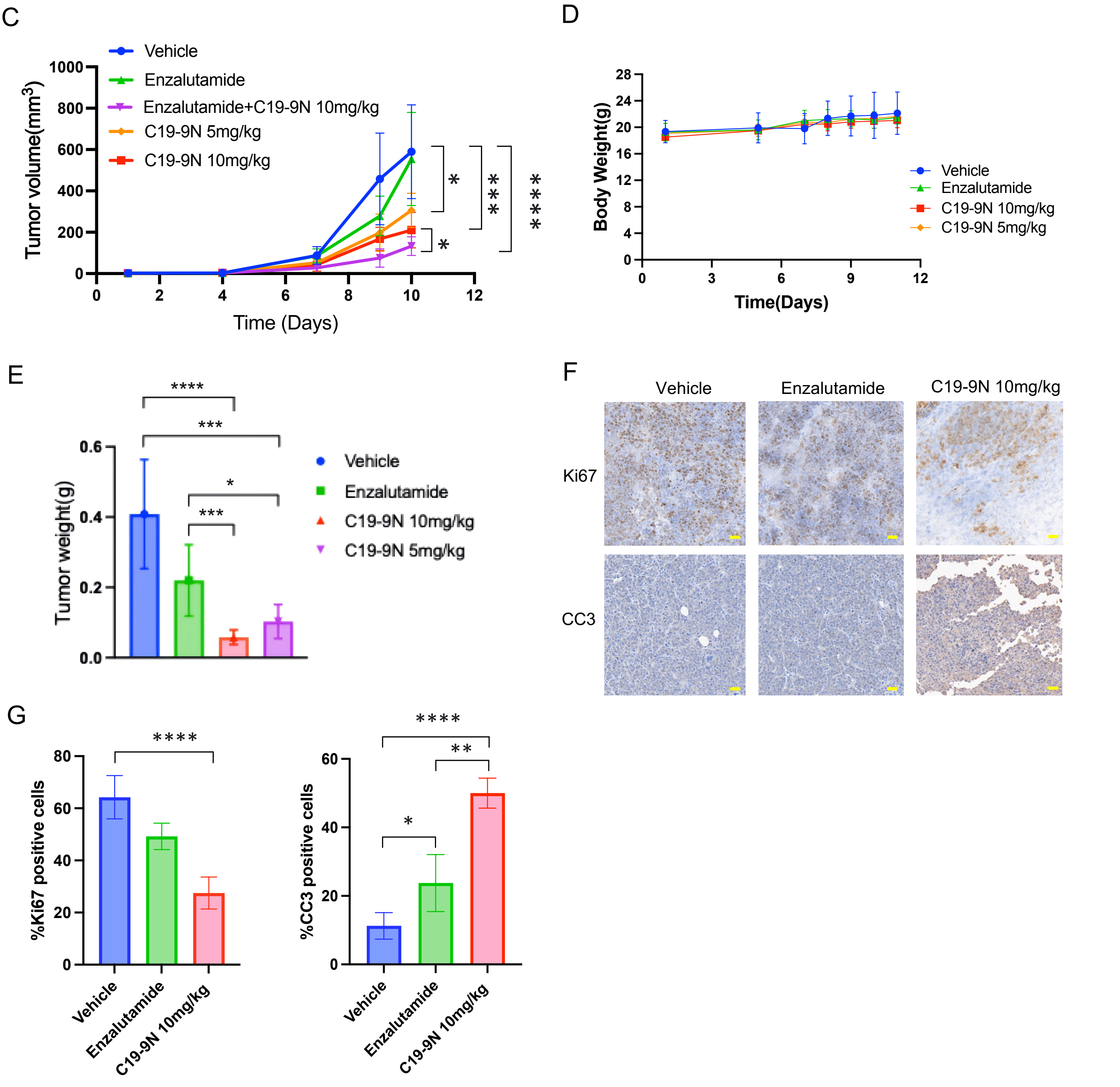

Figure S6. Pharmacokinetic profile and in vivo anti-tumor efficacy of C19-9N in prostate cancer xenograft models.

(A) Pharmacokinetic (PK) parameters of C19-9N.

(B) Body weight stability in TRAMP-C1 xenograft model. Body weight curve of mice treated with vehicle, enzalutamide, enzalutamide with C19-9N (10 mg/kg), C19-9N (5/10 mg/kg) over 10 days.

(C) C19-9N suppresses TRAMP-C1 xenograft growth. Tumor volume growth curve over 10 days.

(D) Body weight stability in 22RV1 xenograft model. Body weight curve of mice treated with vehicle, enzalutamide, C19-9N (5/10 mg/kg) over 12 days.

(E) C19-9N inhibits 22RV1 xenograft progression. Quantitative analysis of tumor weight.

(F) Representative Immunohistochemistry (IHC) images of 22RV1 tumor sections stained for the proliferation marker Ki-67 (top) and the apoptosis marker Cleaved Caspase-3 (CC3) (bottom). Scale bar = 50 μm.

(G) Left: Quantitative analysis of Ki-67-positive cells. Right: Quantitative analysis of CC3-positive cells. Data are expressed as mean ± SD, *P-value < 0.05, **P-value <0.01, ***P-value < 0.001.

Figure S7

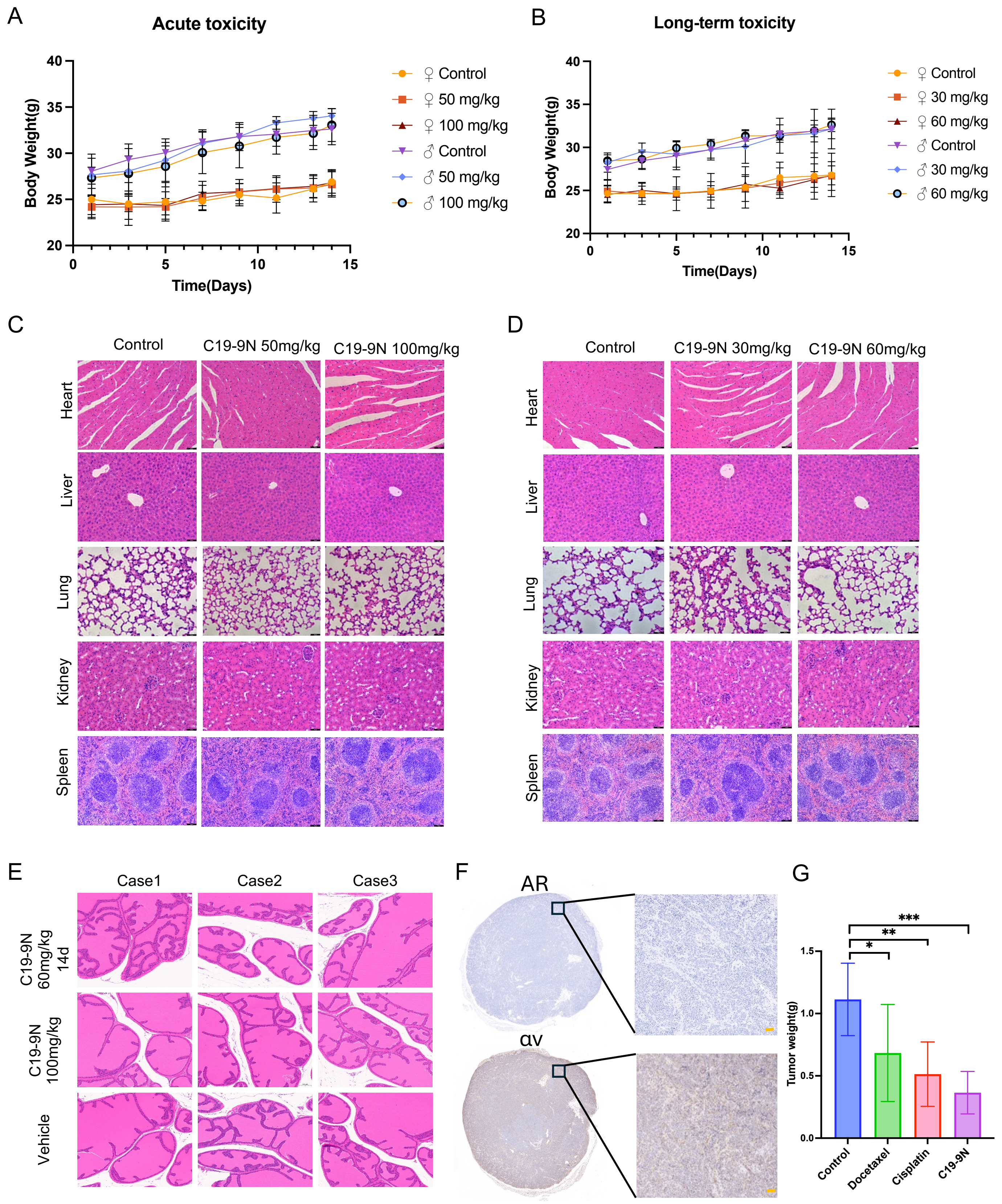
A
Acute toxicity
Body Weight(g)
Time(Days)
♀ Control
♀ 50 mg/kg
♀ 100 mg/kg
♂ Control
♂ 50 mg/kg
♂ 100 mg/kg
B
Long-term toxicity
Body Weight(g)
Time(Days)
♀ Control
♀ 30 mg/kg
♀ 60 mg/kg
♂ Control
♂ 30 mg/kg
♂ 60 mg/kg
C
Control
C19-9N 50mg/kg
C19-9N 100mg/kg
Heart
Liver
Lung
Kidney
Spleen
D
Control
C19-9N 30mg/kg
C19-9N 60mg/kg
Heart
Liver
Lung
Kidney
Spleen
E
Case1
Case2
Case3
C19-9N 60mg/kg 14d
C19-9N 100mg/kg
Vehicle
F
AR
αv
G
Tumor weight(g)
Control
Docetaxel
Cisplatin
C19-9N
*
**
***

Figure S7. Safety evaluation of C19-9N.

(A) Long-term toxicity body weight analysis. Body weight curve of mice (male/female) treated with C19-9N (30/60 mg/kg) vs. control over 14 days (no dose-dependent weight loss).

(B) Acute toxicity body weight analysis. Body weight curve of mice (male/female) treated with C19-9N (50/100 mg/kg) vs. control over 14 days (no acute weight loss).

(C, D) Histopathological evaluation of major organs. Hematoxylin-eosin (H&E) staining of heart, liver, lung, kidney tissues from control or C19-9N-treated (30/60 mg/kg) mice (no overt treatment-related lesions).

(E) Representative Hematoxylin and Eosin (H&E) staining of prostate tissues from mice treated with Vehicle or C19-9N (60 mg/kg and 100 mg/kg) for 14 days. Histopathological examination reveals no significant drug-related toxicity, structural damage, or inflammation in the prostate tissues across the treated groups compared to the control, further supporting the in vivo safety of C19-9N at therapeutic doses.

(F) AR and αv expression in NEPC PDX tumor. Representative IHC staining of AR (Top) and αv (Bottom) in NEPC PDX tumor (AR-negative, αv-high expression). Scale bar = 100 μm.

(G) Tumor weight in NEPC PDX model. Quantitative tumor weight of mice treated with Control, Docetaxel, Cisplatin, C19-9N (20 mg/kg). Data are expressed as mean ± SD, *P-value < 0.05, **P-value <0.01, ***P-value < 0.001.

Figure S8

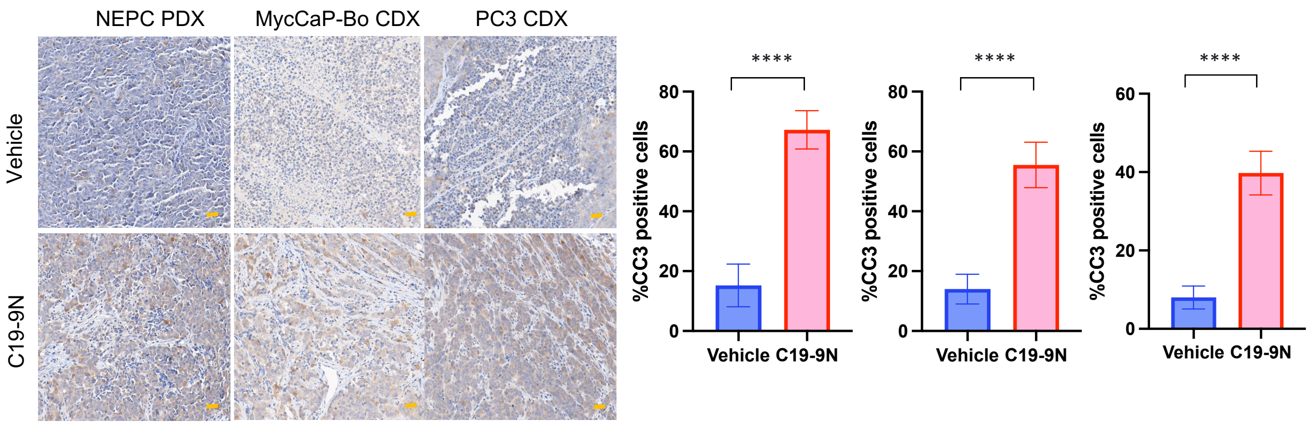

NEPC PDX
MycCaP-Bo CDX
PC3 CDX
Vehicle
C19-9N
****
%CC3 positive cells
0
20
40
60
80
Vehicle
C19-9N
****
%CC3 positive cells
0
20
40
60
80
Vehicle
C19-9N
****
%CC3 positive cells
0
20
40
60
Vehicle
C19-9N

Figure S8. C19-9N induces apoptosis in diverse prostate cancer models. IHC analysis of Cleaved Caspase 3 (CC3) in patient-derived xenograft (PDX) or cell line-derived xenograft (CDX) tumor sections treated with Vehicle or C19-9N. C19-9N treatment significantly increased the percentage of CC3-positive cells in all three models, demonstrating its potent pro-apoptotic activity across different prostate cancer subtypes. Data are expressed as mean ± SD, *P-value < 0.05, **P-value <0.01, ***P-value < 0.001.

Figure S9

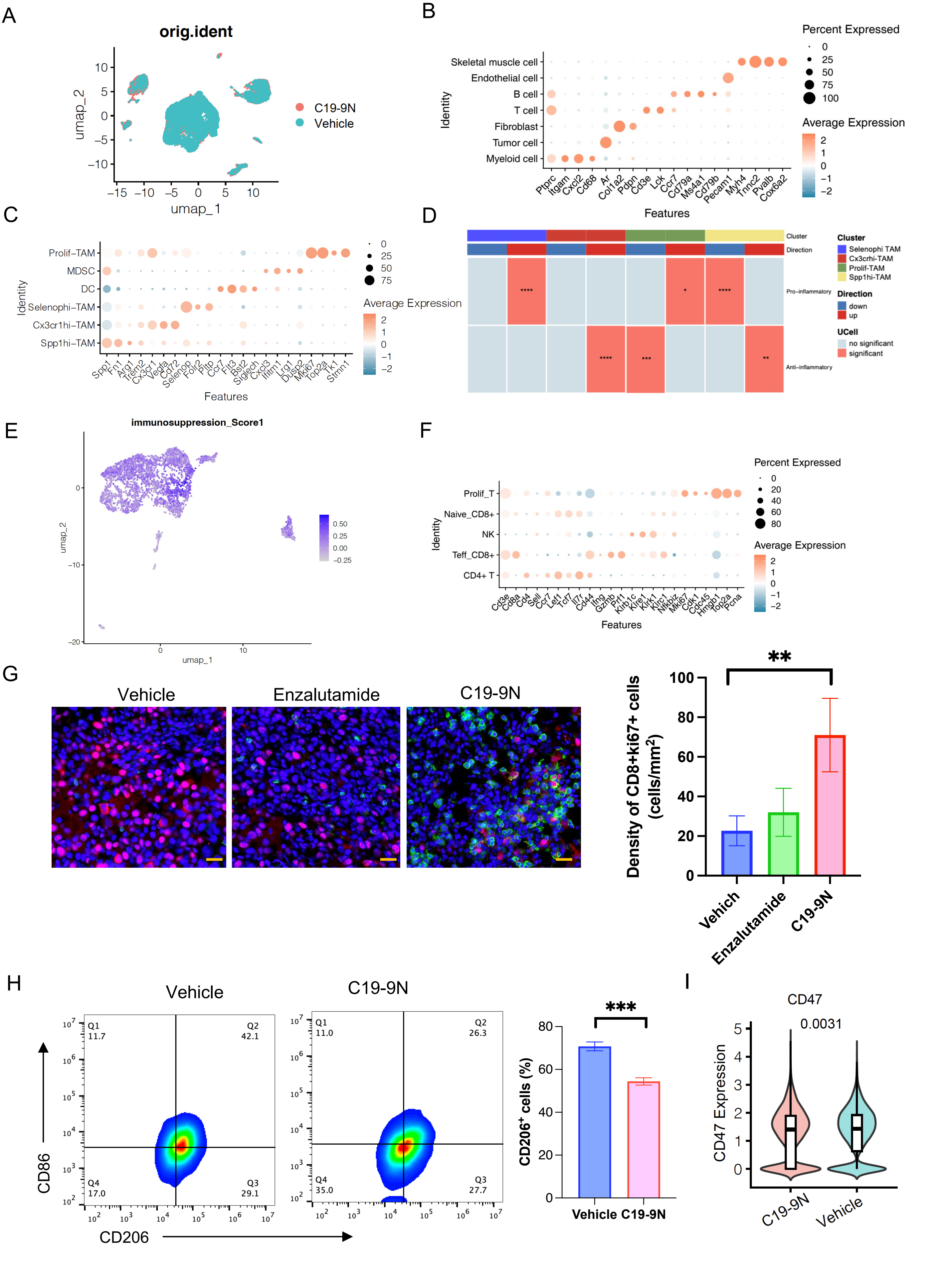
A
orig.ident
C19-9N
Vehicle
umap_1
umap_2
B
Percent Expressed
Average Expression
Skeletal muscle cell
Endothelial cell
B cell
T cell
Fibroblast
Tumor cell
Myeloid cell
Identity
Features
C
Prolif−TAM
MDSC
DC
Selenophi−TAM
Cx3cr1hi−TAM
Spp1hi−TAM
Average Expression
Features
D
Cluster
Direction
Selenophi TAM
Cx3crhi-TAM
Prolif-TAM
Spp1hi-TAM
down
up
UCell
no significant
significant
Pro−inflammatory
Anti−inflammatory
E
immunosuppression_Score1
umap_1
umap_2
F
Prolif_T
Naive_CD8+
NK
Teff_CD8+
CD4+ T
Percent Expressed
Average Expression
Features
G
Vehicle
Enzalutamide
C19-9N
Density of CD8+ki67+ cells (cells/mm2)
Vehich
Enzalutamide
C19-9N
H
Vehicle
C19-9N
CD86
CD206
Q1 11.7
Q2 42.1
Q4 17.0
Q3 29.1
Q1 11.0
Q2 26.3
Q4 35.0
Q3 27.7
CD206+ cells (%)
Vehicle C19-9N
I
CD47
0.0031
CD47 Expression
C19-9N
Vehicle

Figure S9. C19-9N remodels TME immune subsets via TAM polarization and CD47 downregulation.

(A) scRNA-seq grouping of tumor-infiltrating cells.

(B) Cell type-specific gene expression profileDot plot showing average expression (color) and percent expression (dot size) of cell type marker genes across major TME cell subsets.

(C) Myeloid subset-specific gene expression. Dot plot showing marker gene expression (color: average; size: percent) across myeloid subsets.

(D) irGSEA analysis of myeloid subset pathways. Heatmap of irGSEA pathway enrichment scores across myeloid subsets.

(E) Immunosuppression score in TME cells. UMAP plot of TME cells colored by immunosuppression score.

(F) T cell subset-specific gene expression. Dot plot showing marker gene expression (color: average; size: percent) across T cell subsets.

(G) C19-9N increases $CD8^{+}Ki67^{+}$ T cell abundance. Scale bar = 20 μm.

(H) C19-9N reduces CD206+ TAMs in BMDM polarization assays.

(I) Violin plot of C19-9N downregulation of CD47 expression via scRNA-seq analysis in bone metastatic TME (P = 0.0031). Data are expressed as mean ± SD, *P-value < 0.05, **P-value <0.01, ***P-value < 0.001.
