# Supplementary material for: An orally bioavailable pan-αv/α5β1 integrin antagonist prevents aggressive prostate cancer progression via suppressing both oncogenic signals and CD47-mediated immune escape
Source: Mol Cancer. 2026 May 13;25:166. doi: 10.1186/s12943-026-02686-7 (PMC13343748; doi:10.1186/s12943-026-02686-7)

The full uncropped Gels and Blots images

Figure 2E

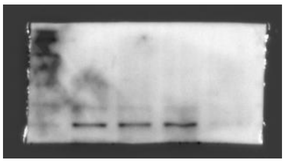

mcl1

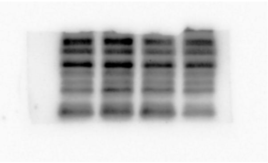

Bcl2

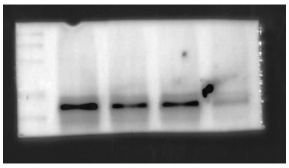

C-myc

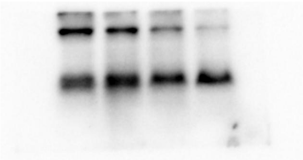

Survivn

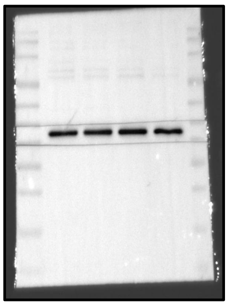

GAPDH

Figure 4A

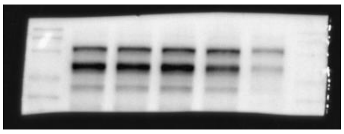

AR

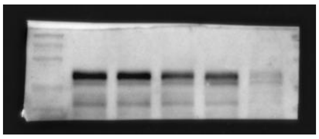

AR-V7

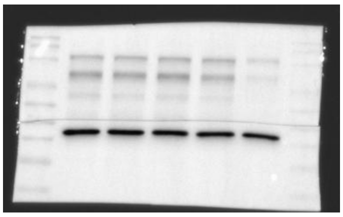

GAPDH

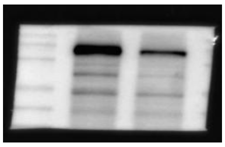

αV

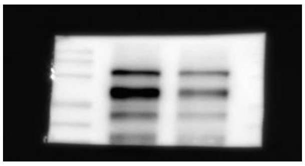

AR

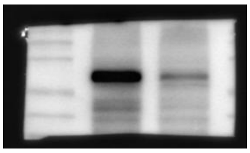

AR-V7

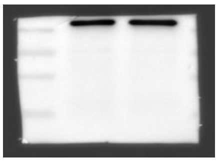

GAPDH

Figure 4B

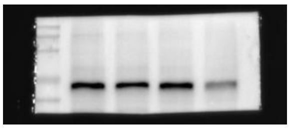

SRC

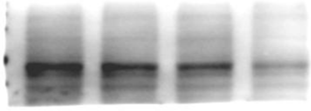

P-SRC

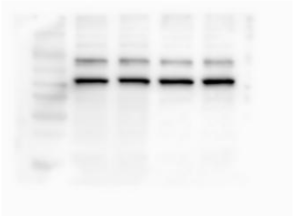

AKT

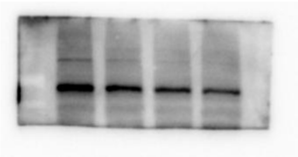

P-AKT

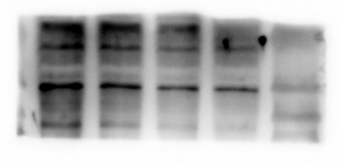

STAT3

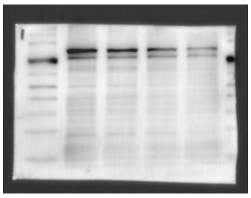

P-STAT3

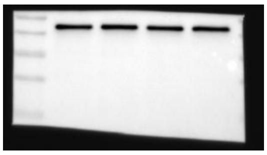

GAPDH

Figure 7G

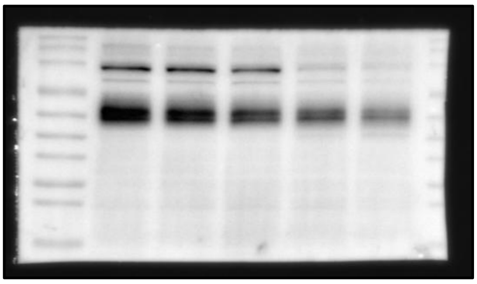

CD47

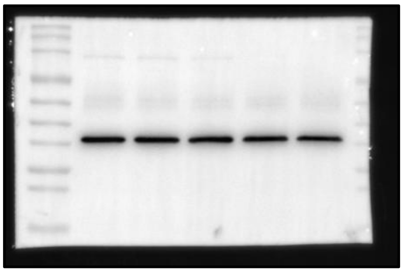

GAPDH

Figure S1B

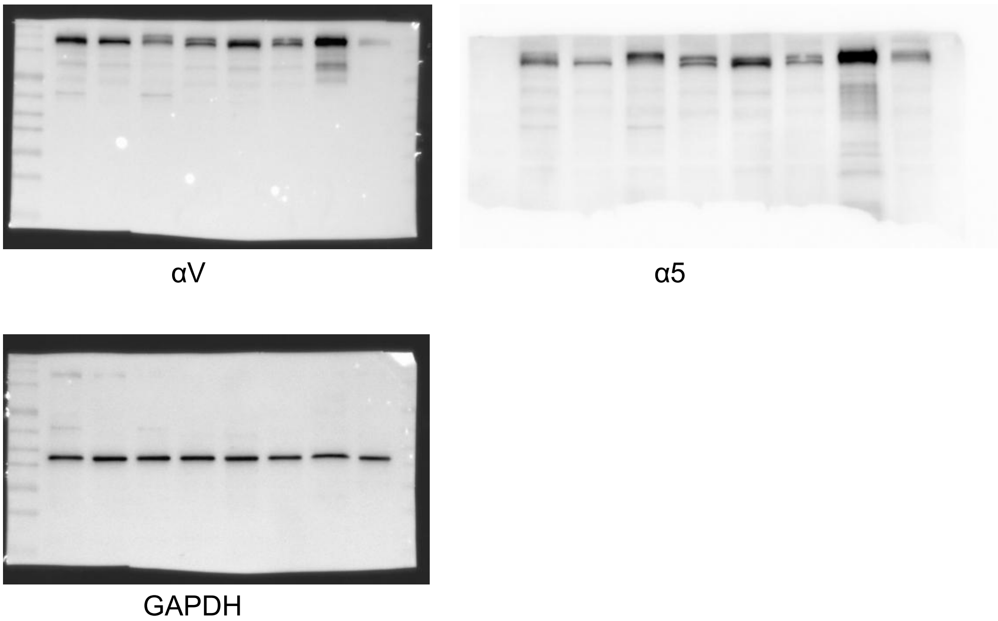

Figure S1C

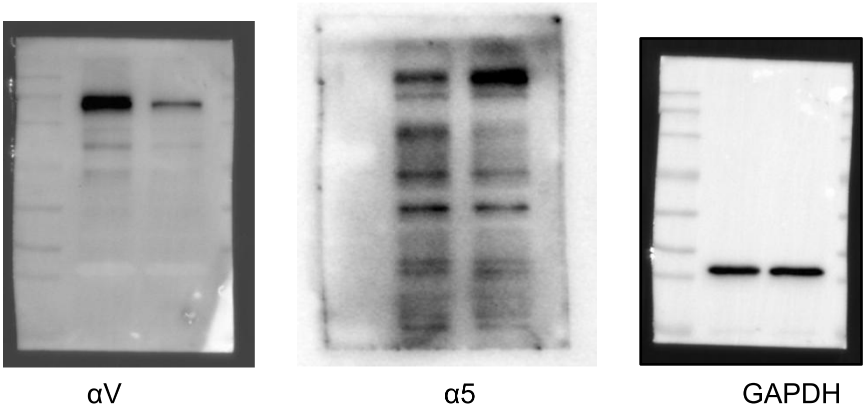

Supplement: Supplementary file 2 — Supplementary Material 2. [file 12943_2026_2686_MOESM2_ESM.pdf]
